# Selected Abstracts from the 27th Annual Meeting of the Society in Europe for Simulation Applied to Medicine: SESAM Virtual Annual Meeting 2022. Seville, Spain, 15-17 June 2022

**DOI:** 10.1186/s41077-022-00211-6

**Published:** 2022-06-15

## SESAM: Improved Healthcare Through Simulation

### Society for Simulation in Europe

#### 0001 A comparison of the psychomotor skills performance between two dental student year cohorts using the Simtocare, haptically enabled, mixed reality simulator

##### Barry F.A. Quinn^1^, Grace Tern^2^, Guneet Kaur Kukureja^2^

###### ^1^University of Liverpool; ^2^King's College London

**Format:** Research Studies - Oral Presentations and Short Communications

**Topic:** Assessment using Simulation

***Introduction***

Haptic simulators allow for virtual-reality-based training and assessment of manual and psychomotor skills needed in operative dentistry, such as spatial awareness and hand-eye coordination.

Previous research (hapTEL study) at King’s Dental Institute revealed an improvement in clinical skills of a single cohort over time when performing a series of different tasks on the simulators. However, there are limited studies that compare progression of clinical performance on haptic simulators across cohorts.

This non-interventional study aimed to investigate differences in psychomotor skills performance characteristics between dental students in clinical years 3 and 5, performing the same task.

***Methods***

Anonymized data were collected retrospectively for an identified common task (Fig 1) taught in years 3 (n=99) and year 5(n=136) from computer logs in the SIMtoCARE Dente training simulators (Fig 2).

Manual Dexterity Task (man08-DONUT) was identified as the common task (Fig 1). The students were instructed to remove the blue coloured volume and try to avoid the orange leeway (0.2mm thick) and the base white colour.

Clinical tutors decided on the parameters of ’clinical acceptability’ that students had to aim to satisfy:

- ≥96% target removal- (blue)
- ≤7% leeway removal (orange)
- <1% container removal (white)

Students were asked to submit their best attempt, with no limit on number of attempts.

***Results & Discussion***

For raw data with no exclusions for “clinical acceptability”, the mean target and leeway scores were calculated (Table 1) After the raw data was filtered by the inclusion criteria (≥96% target ≤7% leeway and <1% container):

1. No student in year 3 fulfilled all 3 parameters, but 27.27% of the students met at least two of three parameters
2. 47.80% of year 5 students met all three parameters.

A clear improvement in the standard of psychomotor skills were demonstrated between years 3 and 5. Encouragingly, the data revealed that there is a potential for students to improve their clinical skills with deliberate practice, as they move through the years. The objectiveness of the feedback afforded the opportunity for more student-centred learning. This study also adds to the evidence-base of the effectiveness of haptic virtual-reality simulators for teaching clinical dental surgical skills.

***Clinical speciality keyword***

Restorative Dentistry, Operative Dentistry,

***References/Acknowledgements***

I wish to thank the King's Undergraduate Research Fund (KURF) for sponsored two of the authors. Reference

Cox M.J.; Quinn B. F.A. & San Diego J. Factors Influencing Students’ Progression and Self-Reporting Practices using Haptic Work-Stations for Learning Basic Clinical Skills. Presented at AERA June 2020

***Ethics Statement***

The authors declare that all procedures followed were in accordance with the ethical standards of the responsible committee on human experimentation (institutional and national) and with the Helsinki Declaration of 1975 (In its most recently amended version).

This study was data mining retrospectively anonymised data from previously timetabled teaching sessions and no intervention occurred. The University ethics committee, stated that no further ethic application was necessary.

**Fig. 1 (abstract 0001).**
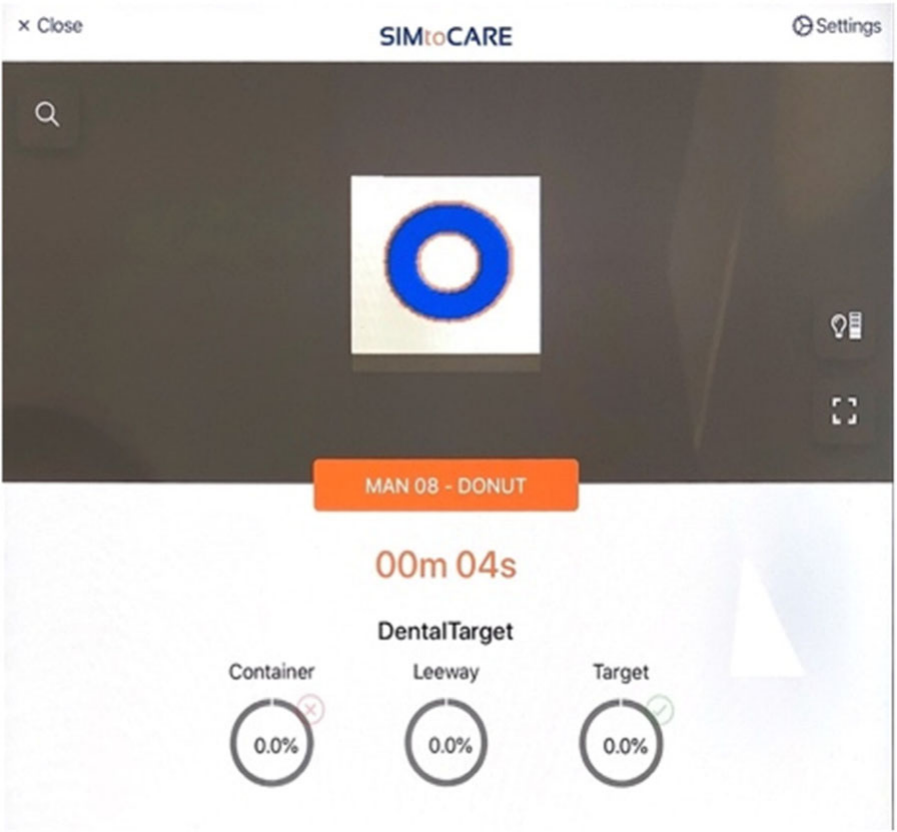
Dough-nut task

**Fig. 2 (abstract 0001).**
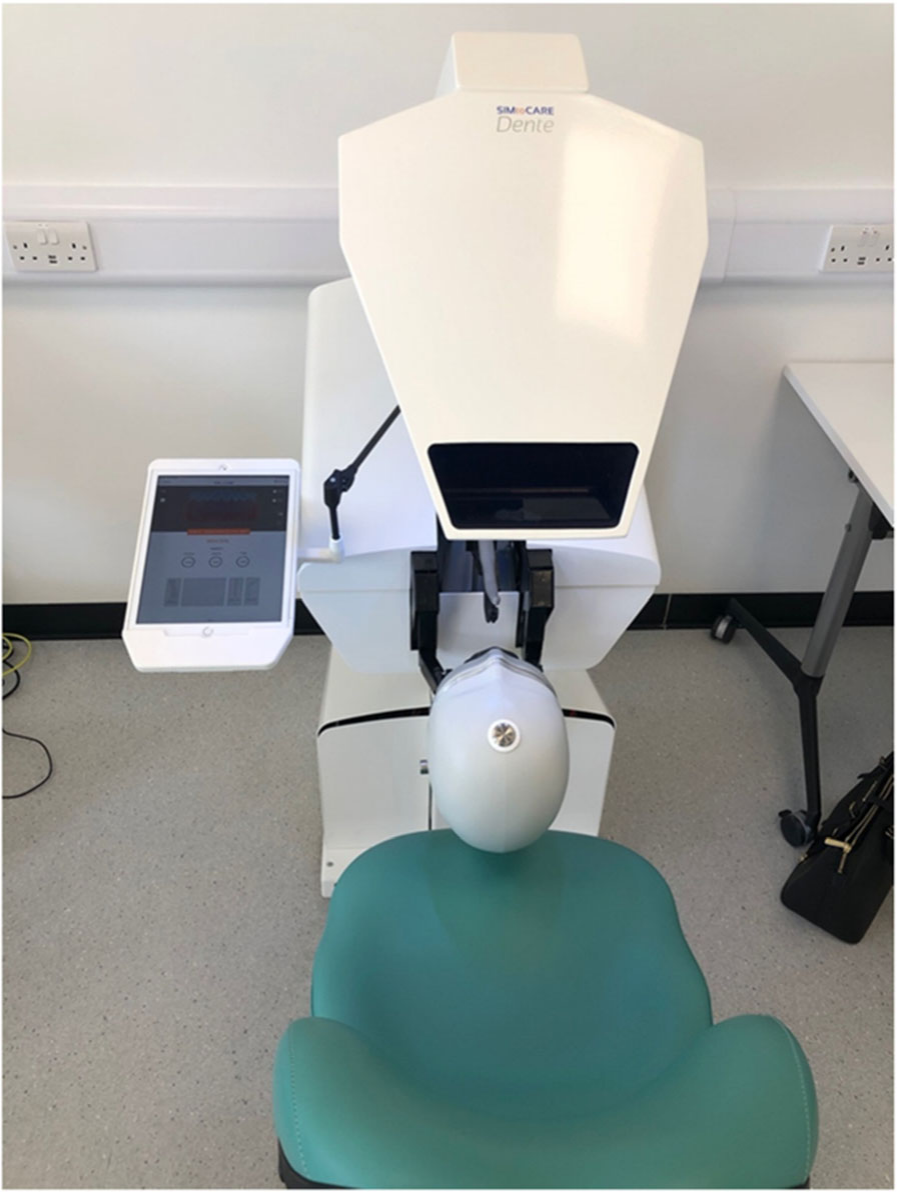
SIMtoCare Dental Simulator

**Table 1 (abstract 0001).** Raw data for target and leeway scores for years 3 and 5

	BDS3 (n=99)	BDS5 (n=136)
Mean target score (%)	90.91	96.34
Mean leeway score (%)	22.71	7.18

#### 0002 ADRIS: A new Accessible DRIving Simulator for people with sensorimotor disability

##### Serena Ricci^1^, Filippo Gandolfi^1^, Giorgia Marchesi^1^, Amy Bellitto^1^, Paolo Romani^1^, Andrea Canessa^1^, Angelo Basteris^2^, Antonino Massone^3^, Maura Casadio^1^

###### ^1^University of Genova; ^2^University of Southern Denmark; ^3^Santa Corona Hospital

**Format:** Research Studies - Oral Presentations and Short Communications

**Topic:** New Technologies and Innovation

***Introduction***

Accessibility is crucial to enable persons with disabilities to live independently. Driving a car is helpful against social disability, albeit complicated for persons with sensorimotor disabilities. Driving simulators offer a mean to practice driving in a safe environment, while maintaining real life challenges. Commercial driving simulators are expensive and voluminous [1]. Also, most of them are not immediately and freely accessible to users with severe motor disability, often lack immersiveness, thus not delivering a fully realistic driving experience, and do not provide driving performance measures. The goal of this study was to design, develop and test a new open-source, highly accessible, realistic virtual reality (VR) driving simulator for people with disability.

***Methods***

Accessible DRIving Simulator (ADRIS) consists of a PC, a driving controller and either a display or immersive VR system (e.g., HTC Vive, Oculus Quest). By adapting an open-source simulator for autonomous driving research (CARLA [2]), developed in the graphic engine Unreal Engine, we implemented ADRIS, which includes scenarios with different difficulties in terms of traffic, road signs, light and weather conditions. The virtual vehicle contains a replica of the driving controller that moves as in reality. To test the system, we collected performance and muscular activity of 17 healthy subjects who underwent a 1-hour session consisting in 3 levels of increasing difficulty preceded and followed by a free driving test. At the end of the experiment, participants filled out 3 questionnaires to assess discomfort [3], task load [4] and overall user experience [5].

***Results & Discussion***

Max speed, speed variability slightly increased with practice, suggesting that users were more familiar and comfortable with the simulator at the end of the session. Speed data also revealed that users were slower than they would be in real life. Possible explanations may be that they were not familiar with the VR application, or that 1-hour of driving is not enough to be comfortable with a new car, as in real life. Questionnaires reported no general discomfort, no motion sickness3, no physical fatigue, a moderate mental effort, no frustration4, and a high level of immersivity and sense of presence5.

In conclusion, ADRIS provides a realistic driving experience in a safe and controlled environment. Many of the scenarios that usually occur while driving can be presented to the user. Also, performance and behaviors can be assessed, thus making ADRIS promising to improve road safety and to help recovering the driving skills of people with disability.

***Clinical speciality keyword***

rehabilitation, driving simulator, driving skills, virtual reality

***References/Acknowledgements***

1. Rodseth J, Washabaugh EP, Al Haddad A, Kartje P, Tate DG, Krishnan C. A novel low-cost solution for driving assessment in individuals with and without disabilities. Appl Ergon. 2017;65:335-344. https://doi.org/10.1016/j.apergo.2017.07.002.

2. Dosovitskiy A, Ros G, Codevilla F, Lopez A, Koltun V. CARLA: An open urban driving simulator. In: Conference on Robot Learning. PMLR; 2017:1-16.

3. Balk SA, Bertola MA, Inman VW. Simulator sickness questionnaire: Twenty years later. 2013.

4. Hart SG, Staveland LE. Development of NASA-TLX (Task Load Index): Results of empirical and theoretical research. In: Advances in Psychology. Vol 52. Elsevier; 1988:139-183.

5. Moss-Morris R, Weinman J, Petrie K, Horne R, Cameron L, Buick D. The revised illness perception questionnaire (IPQ-R). Psychol Heal. 2002;17(1):1-16.

***Ethics Statement***

The study was approved by the Institutional Review Board of the Department of Informatics, Bioengineering, Robotics and Systems Engineering (DIBRIS), University of Genoa, Genoa, Italy (code CE DIBRIS protocol - 009/2020 approved on 18/05/2020) and conformed to the ethical standards of the 1975 Declaration of Helsinki. Each subject provided written informed consent to participate in the study and to publish individual data.

**Fig. 1 (abstract 002).**
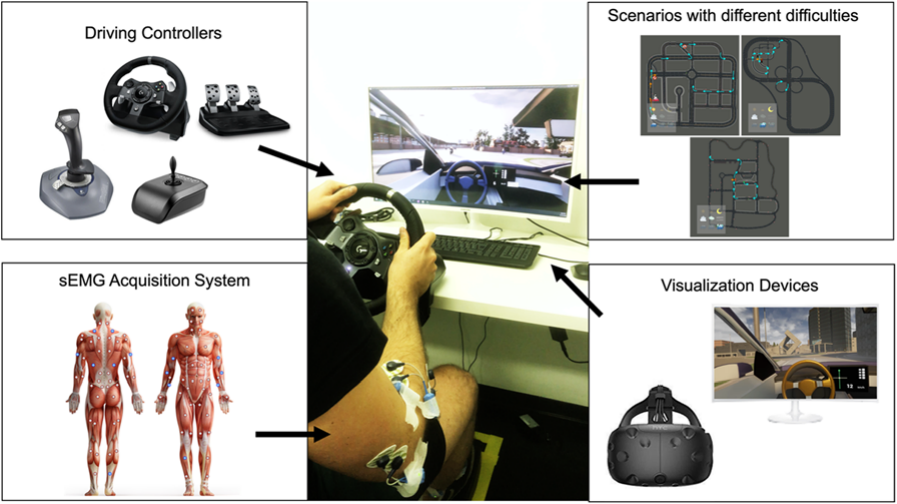
See text for description

#### 0003 Blended learning achivements compared to face-to-face learning achivements in clinical simulation-based teaching

##### Ana Carolina del Pozo, Licia Montagna, Silvia Oldani, Valeriano Vinci, Stefania Brusa

###### Humanitas University

**Format:** Descriptive Work - Oral Presentations and Short Communications

**Topic:** New Technologies and Innovation

***Introduction***

Patient management training is a key goal at Humanitas Medical School. The articulated “Professionalism Activities Program” of our curricula has among its goals to develop student’s technical skills (TS) and non-technical skills (NTS). This Program includes “Simulation-Based teaching” as a powerful facilitator. Because of the current COVID-19 pandemic, the simulation-based learning activities modality switched from face-to-face to blended. This study aims to compare the blended-based learning outcomes with the previous face-to-face learning outcomes and to identify future challenges, and perspectives.

***Description***

Our study compared the 5th year medical student performance during the face-to-face simulation-based learning in 2019 (Face-to-face-group) with the 5th year medical student performance obtained with the blended learning implemented during the COVID-19 pandemic (Blended-group). Each group consisted of 60 medical students that had the same tutor for their clinical simulation-based learning activities, feedback, and assessment. The assessment was identical for the two groups and consisted in the continuous evaluation of student’s TS and NTS through 35 variables measured with a 5-point Likert-scale by the same tutor and analyzed through Wilcoxon signed-rank test.

RESULTS: There was not statistically significant difference between the two groups when TS variables were assessed. Statistically significant differences were found in different NTS variables. Team working (P<0.001), communication with patients and colleagues (P=0.001), adaptation of guidelines to the situation (P<0.001), decision making and situation awareness (P=0.001), connection of gathered information (P=0.013) were significantly reduced in the Blended-group.

***Discussion***

Current COVID-19 pandemic has a dramatic effect on medical education. Blended learning results to be a valid solution to maintain the learning processes. In our study, though, with the blended modality, students did not obtain similar levels of NTS performance as with the face-to-face modality. Better understanding and analysis are needed for programming arrangements to reach through the blended modality this important goal.

Among the several challenges of medical education in this current situation, the development of the NTS needs special effort and reinforcement. With some challenging changes, blended learning approach may not only be a tool for tackling the medical education dilemma during this pandemic but might also serve to define new strategies for teaching activities in the future.

***Clinical speciality keyword***

Medical Education - Patient Management

***References/Acknowledgements***

1. Gandolfi A. (2021). Planning of school teaching during Covid-19. Physica D. Nonlinear phenomena, 415, 132753. https://doi.org/10.1016/j.physd.2020.132753

2. Gintrowicz, R., Pawloy, K., & Degel, A. (2021). Social distancing in advanced emergency medicine courses - can it work?. GMS journal for medical education, 38(1), Doc22. 10.3205/zma001418

3. Singh, K., Srivastav, S., Bhardwaj, A., Dixit, A., & Misra, S. (2020). Medical Education During the COVID-19 Pandemic: A Single Institution Experience. Indian pediatrics, 57(7), 678–679. 10.1007/s13312-020-1899-2

4. Mundell, W. C., Kennedy, C. C., Szostek, J. H., & Cook, D. A. (2013). Simulation technology for resuscitation training: a systematic review and meta-analysis. Resuscitation, 84(9), 1174–1183. https://doi.org/10.1016/j.resuscitation.2013.04.016

5. Mundell, W. C., Kennedy, C. C., Szostek, J. H., & Cook, D. A. (2013). Simulation technology for resuscitation training: a systematic review and meta-analysis. Resuscitation, 84(9), 1174–1183. https://doi.org/10.1016/j.resuscitation.2013.04.016

6. Hartmann, L., Kaden, J. J., & Strohmer, R. (2021). Authentic SP-based teaching in spite of COVID-19 - is that possible?. GMS journal for medical education, 38(1), Doc21. https://doi.org/10.3205/zma001417

***Ethics Statement***

The authors declare that they have followed the guidelines for scientific integrity and professional ethics. The article does not contain any studies with human or animal subjects.

#### 0004 Can simulation-based activities bring Higher Education Institutions and hospitals closer together?

##### Une Elisabeth Stømer, Sigrun Anna Qvindesland

###### Stavanger University Hospital

**Format:** Descriptive Work - Oral Presentations and Short Communications

**Topic:** Assessment using Simulation

***Introduction***

There are several major challenges in the Norwegian hospitals/healthcare system today, for example, lack of competent healthcare professionals due to high turnover, difficulties in providing clinical placements for students, a high drop-out of students during education, and a growing rate of adverse events in hospitals (1).

Simulation-based learning activities (SBLA) as educational methods may contribute to addressing some of these challenges (2). Several publications support the efficacy of using SBLA of high quality in health professions (3-8).

The Western Norway Region Health Authority (Helse Vest) commissioned the regional coordinating unit for SBLA (RegSim Vest) to lead a group of representatives for SBLA from Higher Education Institutions (HEI’s) and hospitals. The purpose was to 1. identify relevant topics for research projects to assess the effects of simulation in education, 2. potential financing, and 3. provide suggestions for how to increase the collaboration between the HEIs and the hospitals in research on the effects of SBLA.

All regional HEIs (four) and hospitals (five) were invited and accepted to participate in the work. The group had two months to collaborate and produce a report to the health authorities.

***Description***

We organized a face-to-face kick-off seminar and four digital meetings. The following processes were undertaken during the period:

- To identify previous and ongoing research projects assessing effects of SBLA in the region, as well as published literature reviews to avoid duplicity, and gauge regional SBLA status.
- To agree on definitions, scope, collaborative principles, and roadmap for research on effects of SBLA in education.
- To produce a framework for research on effects on SBLA in education
- To gather initial, specific research ideas from academics and clinicians
- To suggest relevant topics for research and financing possibilities for future projects in the region, regarding the effects of SBLA in education.

***Discussion***

The group defined education to include both pre-and post-graduates. Further, the group agreed that more research is needed on SBLA as a method (how to perform good SBLA) as well as research that assesses effects on patient outcomes and cost-effivtiveness. Recent published reviews suggest that there is a lack of studies with robust research designs such as Randomized Controlled Trials, multicenter trials, and longitudinal trials (9-11), and is therefore also recommended by the workgroup.

Table 1. Shows an overview of the suggested areas for future research on effects of SBLA in education.

As a closing remark, the working group suggested that the next step is establishing a collaborative SBLA research network between HEI’s and hospitals in writing research protocols, seeking financial support, and performing SBLA projects together to increase the relevance and the quality of the projects.

***Clinical speciality keyword***

N.A.

***References/Acknowledgements***

**References**

1. Jørstad, R. G. (2020). Annual report Norwegian Patient Injury Compensation. Retrieved from https://www.npe.no/globalassets/dokumenter-pdf-og-presentasjoner/arsmelding/2020/arsrapport-2020.pdf

2. Hayden, J. K., Smiley, R. A., Alexander, M., Kardong-Edgren, S., & Jeffries, P. R. (2014). The NCSBN national simulation study: A longitudinal, randomized, controlled study replacing clinical hours with simulation in prelicensure nursing education. Journal of Nursing Regulation, 5(2), S3-S40.

3. Ajmi, S. C., Kurz, M. W., Ersdal, H., Lindner, T., Goyal, M., Issenberg, S. B., & Vossius, C. (2021). Cost-effectiveness of a quality improvement project, including simulation-based training, on reducing door-to-needle times in stroke thrombolysis. BMJ Quality & Safety.

4. Park, C., Grant, J., Dumas, R. P., Dultz, L., Shoultz, T. H., Scott, D. J., . . . Cripps, M. W. (2020). Does simulation work? Monthly trauma simulation and procedural training are associated with decreased time to intervention. Journal of trauma and acute care surgery, 88(2), 242-248.

5. Pilcher, J., Heather, G., Jensen, C., Huwe, V., Jewell, C., Reynolds, R., & Karlsen, K. A. (2012). Simulation-based learning: It’s not just for NRP. Neonatal Network, 31(5), 281-288.

6. Sollid, S. J. M., Dieckman, P., Aase, K., Soreide, E., Ringsted, C., & Ostergaard, D. (2019). Five Topics Health Care Simulation Can Address to Improve Patient Safety: Results From a Consensus Process. J Patient Saf, 15(2), 111-120. doi:https://doi.org/10.1097/PTS.0000000000000254

7. Theilen, U., Fraser, L., Jones, P., Leonard, P., & Simpson, D. (2017). Regular in-situ simulation training of paediatric Medical Emergency Team leads to sustained improvements in hospital response to deteriorating patients, improved outcomes in intensive care and financial savings. Resuscitation, 115, 61-67. doi:10.1016/j.resuscitation.2017.03.031

8. Wisborg, T., Brattebø, G., Brinchmann-Hansen, Å., Uggen, P. E., & Hansen, K. S. (2008). Effects of nationwide training of multiprofessional trauma teams in norwegian hospitals. Journal of trauma and acute care surgery, 64(6), 1613-1618.

9. Heffernan, R., Brumpton, K., Randles, D., & Pinidiyapathirage, J. (2021). Acceptability, technological feasibility and educational value of remotely facilitated simulation based training: A scoping review. Medical Education Online, 26(1), 1972506.

10. PIOT, Marie Aude, et al. Simulation in psychiatry for medical doctors: a systematic review and meta analysis. Medical education, 2020, 54.8: 696-708.

11. Goldshtein, D., Krensky, C., Doshi, S., & Perelman, V. S. (2020). In situ simulation and its effects on patient outcomes: a systematic review. BMJ Simulation and Technology Enhanced Learning, 6(1).

***Ethics Statement***

The authors declare that they have followed the guidelines for scientific integrity and professional ethics. The article does not contain any studies with human or animal subjects.

**Table 1 (abstract 0004).** Thematic overview over suggestions from the workgroup: Research areas on effects of simulation-based learning in education

1) (Pre-graduates) Students’ transition from academic teaching to clinical practice – module-based during pre-graduate education From academic teaching – clinical practice (reduce practice shock) a) preparatory bridge to clinical practice b) replace some clinical practice	
2) (Pre - postgraduates) Transition from pre-graduate – clinical work (reduce school – clinical work shock) - preparation with Simulation Based Learning (SBL)	
3) Continuing Professional Development (CPD): Healthcare professionals’ clinical transitions / professional accountability transitions at work – preparation with SBL	
4) (Post-graduates) SBL as a method in post-graduate formal educations: Nurse Specialists, Residents, etc. achieve learning goals/requirements – quality assurance and exposure assurance	
5) CPD SBL for life-long learning (maintaining competence, improving competence, changes in methods, techniques, procedures / processes)	

#### 0005 CheckList in Trauma Simulation (CheLTS): a new tool for improving trauma management

##### Valerio Teodoro Stefanone^1^, Francesca Innocenti^1^, Irene Rasoini^1^, Federico D’Argenzio^1^, Irene Tassinari^1^, Rita Audisio^1^, Federico Meo^1^, Caterina Savinelli^1^, Giulia Mormando^2^, Riccardo Pini^1^

###### ^1^Azienda Ospedaliero-Universitaria Careggi, Florence, Italy; ^2^Dipartimento di Medicina DIMED, Padova, Italy

**Format:** Research Studies - Oral Presentations and Short Communications

**Topic:** Patient Safety / Quality Improvement

***Introduction***

Trauma is a leading cause of death and disability in the earliest decades of life. Management of major trauma is challenging for emergency physicians due to multiple, simultaneous and potentially fatal lesions. The aim of the present study was to test the effectiveness of a checklist (CL) in improving the management of patients with major trauma.

***Methods***

We tested our hypothesis in a simulation environment. We included 25 teams, each composed by four Emergency Medicine trainees, in which the most expert was the team leader. We designed four scenarios, focused on the management of trauma. The teams managed all the scenarios in a random sequence. We created a CL with the critical actions to be performed in trauma patients. We gave the CL to the teams alternatively during the first or the last two scenarios. The primary outcomes were the adherence to critical processes of care and the time to critical actions in the scenarios managed with CL versus those managed without the CL.

***Results & Discussion***

We identified 52 critical actions, which had to be performed during the simulation. In the scenarios performed with the aid of CL, the number of completed actions was significantly higher than in the scenarios without CL (27±9 vs 24±7 critical actions, p <0.001). By restricting the analysis to critical actions relating to the primary survey, this result was confirmed (22±5 vs 19±4 critical actions, p <0.001). Analyzing the individual actions, in 7 cases they were performed significantly more often in the scenarios performed with the help of the CL: evaluation and treatment of external-back-perineum haemorrhages, removal of all clothes, evaluation of body temperature, immobilization of the cervical spine, evaluation of the neurological status of the 4 limbs (Table 1). As regards the timing of execution of critical actions, among the 49 evaluable actions, a significant reduction in time was observed for only 4 items, in favor of scenarios without checklist: objective examination of the chest, positioning of two venous accesses, sample collection for blood count and coagulation, evaluation of the pulses (Table 1). In conclusion, in a high-fidelity simulation environment, the use of a checklist has improved the completeness of management of the patient with major trauma in the face of a slowdown in execution. These results suggest that the use of a checklist is an important tool for improving patient safety but that its use, still not widespread in clinical practice, requires specific training.

***Clinical speciality keyword***

Emergency Medicine

***References/Acknowledgements***

[1] g. e. al, «Assessment and Resuscitation in Trauma Management,» Surg Clin North Am. , vol. 97, pp. 985-998., 2017 Oct;.

[2] W. D. H. M. e. a. Leape LL, «Promoting patient safety by preventing medical error.,» JAMA , p. 280:1444–1447 , 1998.

[3] J. G. M. L. e. a. Gruen RL, « Patterns of errors contributing to trauma mortality: lessons learned from2594 deaths.,» Ann Surg, p. 244:371–380 , 2006.

[4] H. D. M. M. e. a. Davis JW, « An analysis of errors causing morbidity and mortality in a trauma system: a guide for quality improvement.,» J Trauma, p. 32:660–665, 1992.

[5] S. S. S. G. Y. S. Oakley E, «Using video recording to identify management errors in pediatric traumaresuscitation.,» Pediatrics, p. 117:658–664, 2006.

[6] G. A., The checklist manifesto: how to get things right., New York: Metropolitan Books, 2010.

***Ethics Statement***

The authors declare that they have followed the guidelines for scientific integrity and professional ethics. The article does not contain any studies with human or animal subjects.

**Table 1 (abstract 0005).** See text for description

Critical Action (Yes vs No)	With CL	Without CL	P
Assessment/treatment of external Haemorrages	45 (90%)	36 (72%)	0,041
Assessment/treatment of head Haemorrages	28 (56%)	15 (30%)	0,015
Assessment/treatment of perineum Haemorrages	23 (46%)	8 (16%)	0,002
Cervical Spine Immobilization	31 (62%)	18 (36%)	0,016
Four limbs neurological evaluation	19 (38%)	6 (12%)	0,006
Removal of all clothing	46 (92%)	34 (68%)	0,006
Body temperature assessment	41 (82%)	30 (60%)	0,028
Critical Action time of execution	With CL	Without CL	P
Positioning of two venous accesses	02:37	01:18	0,010
Thorax physical examination	01:34	01:18	0,044
Pulses assessment	04:50	02:56	0,014
Blood exam collecting	04:23	02:40	0,013

#### 0006 Clinicians Standing Positions in Simulated Paediatric Emergencies

##### Phillip Ross, Andrew Thompson, Thomas Bourke, Ben McNaughten, Clare Murray

**Format:** Descriptive Work - Oral Presentations and Short Communications

**Topic:** Interprofessional / Team Education

***Introduction***

Paediatric life support courses have historically focused on clinical knowledge and technical skills however it is now being increasingly recognised the importance of covering leadership and team working skills. The team leader in resuscitation scenarios is now frequently taught they should stand back when managing a critically ill child and not become involved with hands on tasks. We aimed to discover where clinicians from a range of specialties stood during a simulated paediatric emergency and if their standing position resulted in different performance outcomes.

***Description***

Methods

Twenty-seven clinicians from different clinical areas within a tertiary children’s hospital undertook a standardised, six minute, high fidelity simulated paediatric emergency. We recorded standing positions during the scenario and compared the behaviour according to professional background. The time taken to key clinical interventions was also recorded.

Results

There was noticeable difference in standing positions according to professional background (figure 1) ED consultant spent 72% at their time at the end of the bed. PICU consultants spent 36% spent of their at the head of the bed while consultant paediatrics spent 63% standing at the right side and trainees spent similar time in all zones.

PICU consultants were quickest to perform key clinical interventions, followed by ED Consultants, trainees with consultant paediatricians performing least well.

***Discussion***

Clinicians In time critical emergencies adopt different standing positions depending on their training. The best performing consultants (PICU & ED) spent most of their time at the head and the foot of the bed respectively. Further research is needed to evaluate its potential use as an educational tool in the resuscitation setting. This small study supports whilst standing positons are only one of the many factors which resulted in better performance we believe this small study training in acute paediatrics emergencies should emphasise value of standing back and taking an overview of the situation.

***Clinical speciality keyword***

Paediatrics

***References/Acknowledgements***

N/A

***Ethics Statement***

The authors declare that they have followed the guidelines for scientific integrity and professional ethics. The article does not contain any studies with human or animal subjects.

**Fig. 1 (abstract 0006).**
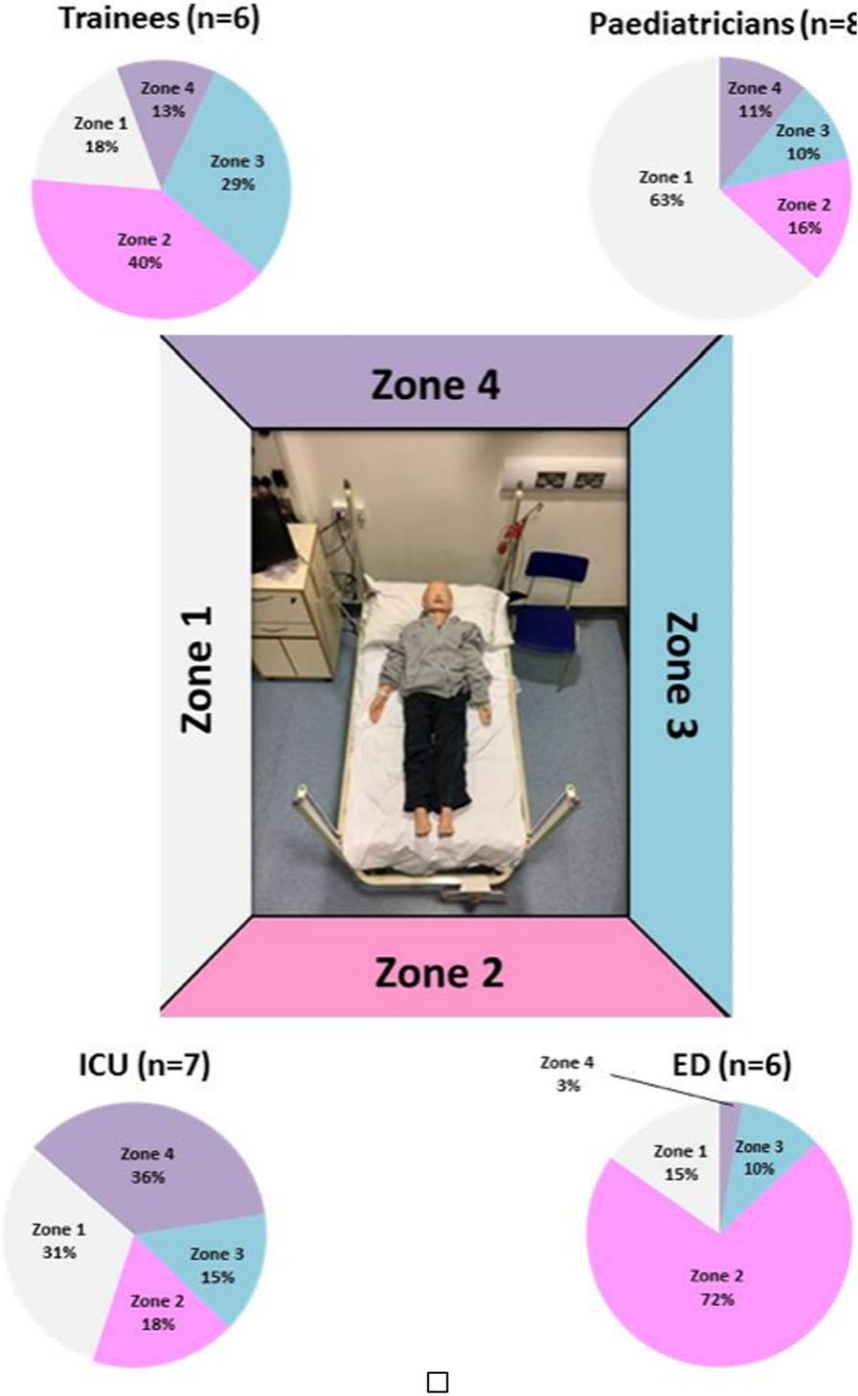
See text for description

#### 0007 Collecting validity evidence for an app-based assessment of cognitive skills for chest tube insertion

##### Leander De Mol^1^, Joris Vangeneugden^2^, Liesbeth Desender^1^, Isabelle Van Herzeele^1^, Lars Konge^3^, Wouter Willaert^2^

###### ^1^Ghent University; ^2^Faculty of Medicine and Health Sciences, Ghent University; ^3^University of Copenhagen

**Format:** Research Studies - Oral Presentations and Short Communications

**Topic:** Assessment using Simulation

***Introduction***

Educational assessments must be validated prior to their implementation. Touch Surgery™(Digital Surgery LTD, London, UK) is a medical simulation application, offering users a learn and test mode to improve and test procedural knowledge. Validity evidence for the chest tube insertion (CTI) test mode was collected using Messick’s contemporary framework.

***Methods***

Novice, intermediate and experienced participants provided informed consent and demographic information. After familiarization, the CTI test mode, consisting of multiple-choice questions, was completed. The resulting percentage score was recorded. Validity evidence was collected from four sources: content, response process, relation to other variables, and consequences. Intermediate and experienced participants completed a post-test questionnaire assessing perceived realism, relevance, and utility of the assessment. Response process was ensured by providing all users with identical familiarization and instructions. Mean scores of the three groups were compared. A pass/fail score was established using the contrasting groups’ method.

***Results & Discussion***

Twenty-five medical students, 11 junior surgical residents, and 19 experienced surgeons participated. Content evidence was collected by an experienced surgeon in CTI and was based on published guidelines and existing literature.

Furthermore, most respondents rated the simulation as realistic, and suitable to assess cognitive skills. Novices scored significantly lower (55.9±7.5) than intermediate (80.6±4.4) (p<0.001) and experienced participants (82.3±5.3) (p<0.001). There was no significant difference between intermediate and experienced participants (p=0.75).

Consequences evidence showed that a pass/fail score of 71% resulted in one false positive (novice that passed) and no false negatives (experienced that failed).

The implementation of this application in surgical curricula was positively reviewed.

The CTI test mode presents a robust validity argument and can be implemented in surgical curricula to assess learners’ cognitive skills prior to hands-on simulation practice. Future investigation concerning internal structure (i.e. reliability) of the assessment is advised.

***Clinical speciality keyword***

Thoracic Surgery

***References/Acknowledgements***

The authors would like to thank Dr. Rafael Grossmann and Joris Vangeneugden for their contributions.

***Ethics Statement***

The authors declare that all procedures followed were in accordance with the ethical standards of the responsible committee on human experimentation (institutional and national) and with the Helsinki Declaration of 1975 (In its most recently amended version).

Written informed consent was obtained from all participants included in the study, and the study was approved by the Ethics Committee of Ghent University Hospital.

**Fig. 1 (abstract 0007).**
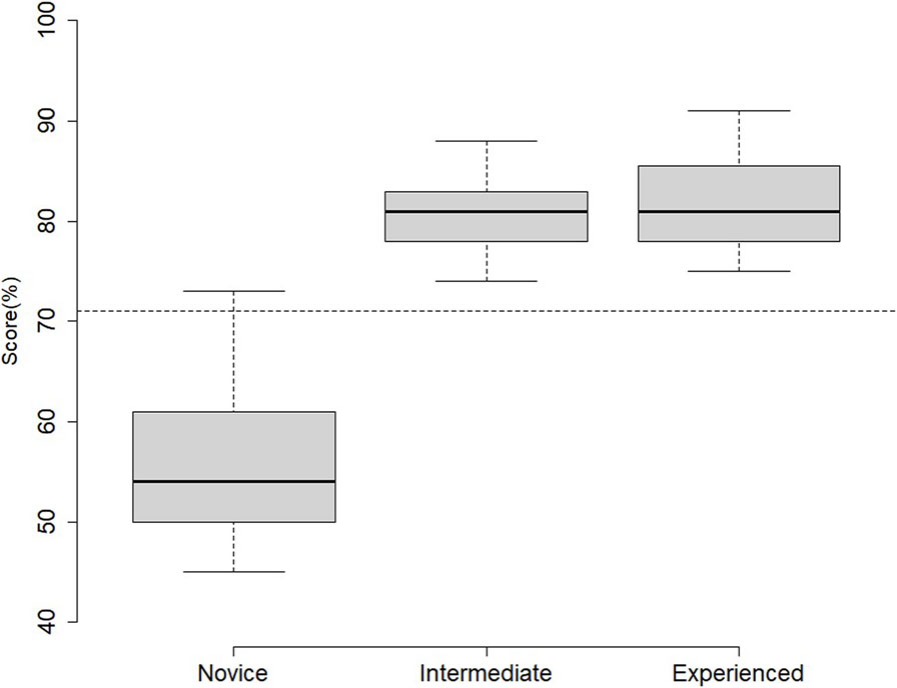
See text for description

#### 0008 COVID as a Catalyst: Improving Undergraduate Paediatric Emergency Simulation Training

##### Assim Javaid^1^, Thomas Cromarty^2^, Natasha Jones^3^, Eunice TK Pak^3^, Saiyanora Hemrom^1^, Siva Oruganti^4^

###### ^1^Cardiff University; ^2^Queen Alexandra Hospital; ^3^School of Medicine; ^4^Noah's Ark Hospital

**Format:** Descriptive Work - Oral Presentations and Short Communications

**Topic:** Covid-19

***Introduction***

Cardiff University medical students all take part in paediatric emergency simulation days in Year 4. Due to the COVID19 pandemic virtual simulation sessions were developed and ran through 2021-22. Extensive qualitative feedback was obtained from the medical students over the course of the year. As we move out of the pandemic, and face-to-face simulations are re-introduced, the feedback provided has been used to alter the simulations going forward.

***Description***

During the pandemic, pre-recorded, semi-scripted virtual simulations were used to teach students about medical emergencies. The videos were designed to touch upon key clinical learning points and human factor teaching to be incorporated in the videos. Students from the 2020-21 Year 4 cohort were encouraged at the end of the sessions to fill in an online feedback form.

Data were collected from 200 out of 305 students, following three sessions delivered between November 2020 and April 2021. The feedback form used mixed methods to gather data about their impressions of the virtual simulation day, including 5 open text questions, which allowed for qualitative data to be gathered. The responses to these underwent thematic analysis. Themes were divided into those that showed either advantages or disadvantages of virtual simulation compared to face-to-face simulation. Advantages included more in-depth discussions, increased student confidence, greater convenience and being more pandemic appropriate. Disadvantages included the lack of hands-on experience, technical issues, relative low fidelity and zoom fatigue.

***Discussion***

Face-to-face simulation training has now been re-introduced. However, it has undergone changes following lessons learned from the year of virtual simulation teaching. The main change has been the introduction of a pre-simulation day learning package. This aims to cover a lot of the basic knowledge needed to successfully run a paediatric emergency and is aimed at reducing the cognitive burden on students, thereby increasing their confidence and freeing up more time for discussions.

Alongside this, though the worst of the pandemic seems to have passed, there are still widespread absences on the course due to either having COVID19, being in contact with someone with COVID19 and awaiting test results, or having possible symptoms and awaiting test results. To offer this cohort of students support, the virtual scenarios developed last year are run alongside face-to-face scenarios, so as students can attend virtually if unable to attend face-to-face. Feedback will be collected again form this year’s cohort to allow for continuous development of our paediatric emergency training.

***Clinical speciality keyword***

Paediatrics, pediatrics, paediatric emergency medicine, pediatric emergency medicine

***References/Acknowledgements***

We would like to acknowledge Cardiff University for their support in running these simulations

***Ethics Statement***

The authors declare that all procedures followed were in accordance with the ethical standards of the responsible committee on human experimentation (institutional and national) and with the Helsinki Declaration of 1975 (In its most recently amended version). Informed consent was obtained from all participants included in the study. The project was discussed with the medical education research team at Cardiff University and was deemed to be a “service evaluation”, not “research”, and, as such, was not in need of ethics board approval.

#### 0009 Development and evaluation of a graduate surgical skills curriculum: How to make medical simulation data Findable, Accessible, Interoperable and Reusable

##### Frank Halfwerk^1^, Connie Clare^2^, Erik Groot Jebbink^3^, Marleen Groenier^4^

###### ^1^MST Thoraxcentrum Twente / University of Twente; ^2^Delft University of Technology; ^3^Rijnstate Hospital / University of Twente; ^4^University of Twente

**Format:** Research Studies - Oral Presentations and Short Communications

**Topic:** Curriculum Development

***Introduction***

Increasingly more simulation studies are published under the Open Access publishing model making them freely accessible online to everyone. Often, the only aspect that is not yet open are the underlying datasets from these publications. Publishing datasets improves reproducibility and reliability of research, it increases visibility of research, and accelerates innovation. Furthermore, unique and highly valuable data from i.e. simulation-based training or surgical techniques is not available to everyone.

Our aim is to present a best practice for publishing medical simulation data. A study on development and evaluation of a proficiency-based and simulation-based surgical skills training for technical medicine students is used as an example.

***Methods***

A four-station procedural assessment was developed of basic surgical tasks that included scrubbing and donning, local anaesthesia, incision/excision, and suturing. Performance indicators were determined by an expert panel consisting of four professors in surgery and two technical physicians in surgery. A rubric was developed for scrubbing and donning and procedure-specific rating scales were developed for local anaesthesia, incision/excision, and suturing. The surgical skills training was evaluated after at least one clinical rotation with an online survey.

Data is published according to the FAIR principles: Findable, Accessible, Interoperable and Reusable. To be ‘Findable’, a unique digital object identifier (DOI) was assigned to the dataset, and metadata described the content, contact information, location, items and definitions. The data repository is indexed by search engines, i.e. Google Scholar. The data is ‘Accessible’ for everyone under Open Access. To be ‘Interoperable’, MeSH standards were used. Finally, to be ’Reusable’, the data were made readable by translating and describing the assessment scoring rubrics, addition of documentation, and a license permitting data reuse was assigned.

***Results & Discussion***

Data for 116 master students from two academic years were refined, and student and assessor data anonymised. Age information was grouped by age intervals, so it can be openly published in an external repository. The dataset was made publicly available in the 4TU.ResearchData repository for reuse in i.e. SESAM community. Researchers should be attributed when data is reused under a CC-BY-SA licence.

For medical simulation studies, it is feasible to publish data alongside Open Access peer-reviewed journal articles. The FAIR principles for data management should be incorporated in the design and implementation of future simulation studies.

***Clinical speciality keyword***

Surgery

***References/Acknowledgements***

The authors gratefully acknowledge the Noun Project for the Figure icons: “find” by Adrien Coquet, “context” by Nithinan Tatah, “padlock” by Fahmihorizon, “Recycle” by sripfoto.

Underlying study: Halfwerk, F., Groot Jebbink, E., & Groenier, M. (2020). Development and Evaluation of a Proficiency-based and Simulation-based Surgical Skills Training for Technical Medicine Students. MedEdPublish, 9(1), [3523], https://tinyurl.com/Halfwerk2020

***Ethics Statement***

The authors declare that all procedures followed were in accordance with the ethical standards of the responsible committee on human experimentation (institutional and national) and with the Helsinki Declaration of 1975 (in its most recently amended version). Informed consent was obtained from all participants included in the study. All institutional and national guidelines during the study period (2015-2018) were followed. These specifically excluded projects from requiring ethical approval. Post-hoc ethical review from the Natural Sciences and Engineering Sciences Ethics Committee from the University of Twente stated having no ethical concerns regarding this research (Dated: 20200923).

**Fig. 1 (abstract 0009).**
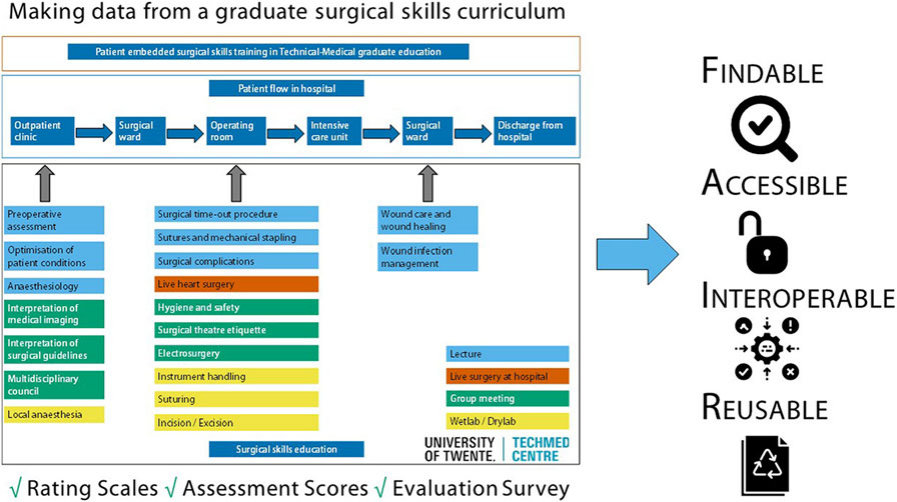
See text for description

#### 0010 Development of simulation-based curriculum to support refugee doctors’ integration into clinical practice in Scotland

##### Julie Doverty^1^, Julie Mardon^1^, Patricia Livingston^2^, Victoria Tallentire^1^, Nisreen Eltom^2^, Sezen Ingilok^1^, Joanne Kerins^1^

###### ^1^Scottish Centre for Simulation and Clinical Human Factors, Forth Valley Royal Hospital; ^2^Dalhousie University

**Format:** Descriptive Work - Oral Presentations and Short Communications

**Topic:** Curriculum Development

***Introduction***

For refugee doctors in the United Kingdom (UK), the path to obtaining full licence for healthcare practice by the General Medical Council (GMC) is arduous. Eligibility for GMC license mandates success in several exams and completion of clinical attachments.

Challenges are further magnified for refugees who may have left their country on short notice without documentation or references, having experienced conflict and war, and possibly having spent years away from clinical practice (1,2). The lengthy legal process to seek refugee status results in prolonged periods of unemployment that erode skills and confidence (3).

Doctors who are refugees are more likely to be referred to the GMC for disciplinary action than their UK born cohorts (4). The GMC “Fair to Refer” project found the root causes of these disproportionate referrals to include inadequate preparation for transitioning to a new cultural and professional environment, lack of clear, constructive feedback, isolation and limited support.

Simulation-based education (SBE) is a highly effective learning technique both for technical and non-technical skills (NTS), offering vivid, experiential learning and reflection (5). Simulation holds potential to complement existing training schemes by providing training in NTS (e.g., communication, cultural practices, decision making) that have been identified as challenges for the refugee cohort.

***Description***

Eighteen refugee doctors are participating in a SBE pilot programme running from October 2021-July 2022. An international curriculum design team with experience in simulation was created to prepare a bespoke SBE programme. The refugee cohort is heterogenous with diverse learning needs. Action research methodology is being employed to optimise curriculum development (6). Initial learning objectives around NTS, the National Health Service, and the roles and responsibilities of a GMC licensed doctor have been identified. Scenario development is ongoing and iterative refinements will occur.

***Discussion***

Our curriculum addresses the issues highlighted by the GMC “Fair to Refer” report. The programme aims to provide a reflective and supportive learning environment that empowers the refugee doctors to integrate more effectively in their future healthcare roles. The curriculum design team considers that improvement in NTS is always possible, and that skills such as communication play a significant role in successful cultural adaptation and safe healthcare. For this reason, NTS are the primary focus of the curriculum. Learning from this curriculum design process and the experience of this cohort has potential application to other settings where refugee or international doctors are being integrated into new healthcare systems.

***Clinical speciality keyword***

Simulation Based Education, Refugee doctors, Action Research

***References/Acknowledgements***

1. Cheeroth S, Goraya A. Refugee doctors. BMJ. 2000 Oct 21;321(7267): S2–7267.

2. Adams K, Borman E. Helping refugee doctors: The new asylum act may make it easier to help small numbers of refugee doctors. BMJ. 2000 Apr 1;320(7239):887–8.

3. Huijskens EGW, Hooshiaran A, Scherpbier A, Horst FVD. Barriers and facilitating factors in the professional careers of international medical graduates. Med Educ. 2010 Aug 1;44(8):795–804.

4. Fair to refer? [Internet]. [cited 2021 Jul 26]. Available from: https://www.gmc-uk.org/about/what-we-do-and-why/data-and-research/research-and-insight-archive/fair-to-refer

5. Khan K, Pattison T, Sherwood M. Simulation in medical education. Med Teach. 2011; 33:1

6. Otto A & Nkanga S (1995) Doing Educational Research: an action research approach, Educational Action Research, 3:3, 279-286,

***Ethics Statement***

The authors declare that they have followed the guidelines for scientific integrity and professional ethics. The article does not contain any studies with human or animal subjects

#### 0011 Effect of Curricular Simulation-based Surgical Training on Proficiency and Patient Outcomes: The SIMULATE Randomised Controlled Trial

##### Abdullatif AYDIN^1^, Kamran AHMED^1^, Takashige ABE^2^, Nicholas RAISON^1^, Mieke VAN HEMELRIJCK^1^, Hashim AHMED^3^, Oliver BRUNCKHORST^1^, Nobuo SHINOHARA^2^, Wei ZHU^4^, Guohua ZENG^4^, John SFAKIANOS^5^, Mantu GUPTA^5^, Ashutosh TEWARI^5^, Ali Serdar GÖZEN^6^, Jens RASSWEILER^6^, Andreas SKOLARIKOS^7^, Thomas KUNIT^8^, Thomas KNOLL^9^, Felix MOLTZAHN^10^, George THALMANN^10^, Andrea LANTZ POWERS^11^, Ben CHEW^12^, Muhammad Shamim KHAN^13^, Prokar DASGUPTA^1^

###### ^1^King's College London; ^2^Hokkaido University Graduate School of Medicine; ^3^Imperial College London; ^4^First Affiliated Hospital of Guangzhou Medical University; ^5^Icahn School of Medicine at Mount Sinai; ^6^SLK Kliniken, University of Heidelberg; ^7^Sismanoglio Hospital, National and Kapodistrian University of Athens; ^8^Paracelsus Medical University; ^9^Klinikum Sindelfingen-Böblingen, University of Tübingen; ^10^University of Bern; ^11^Dalhousie University; ^12^University of British Columbia; ^13^Guy's and St Thomas’ NHS Foundation Trust

**Format:** Research Studies - Oral Presentations and Short Communications

**Topic:** Patient Safety / Quality Improvement

***Introduction***

Simulation-based surgical training is hypothesised to enhance progression along the initial phase of the learning curve. Residents can acquire skills outside of the operating room (OR), without endangering patient safety. However, to date, the transferability of simulation to the OR has been limited to small-scale studies conducted with medical students. The aim of this multicentre randomized controlled trial (ISCRTN 12260261) is to evaluate the effectiveness of simulation training, compared to conventional training in terms of proficiency and patient outcomes.

***Methods***

This international, multicentre randomised controlled superiority trial recruited urology residents(n=94) who had performed ≤10 ureterorenoscopy (URS) cases, as a selected index procedure, with no prior simulation experience. Recruits were randomised to simulation-based training (SBT) or non-simulation- based training (NSBT) groups, the latter of which is the current standard of training. Training sessions were conducted for the SBT arm, utilising an expert-developed multi-modality training curriculum. The primary outcome was the number of procedures required to achieve proficiency, defined as achieving a score of ≥28 on an Objective Structured Assessment of Technical Skill (OSATS) assessment scale, on 3 consecutive operations, without complications. Secondary outcomes included number of surgical complications and stone-free status in each arm. All participants were followed up for 25 procedures or over 18 months.

***Results & Discussion***

A total of 1140 cases were performed by 65 participants where proficiency was achieved in 21 simulation and 18 conventional participants over a median of 8 and 9 procedures, respectively (HR: 1.41 [95% CI 0.72-2.75]). More participants reached proficiency in the simulation arm in flexible ureterorenoscopy, requiring fewer number of procedures (HR 0.89 [95% CI 0.39-2.02]). Significant differences were observed in overall comparison of OSATS scores between groups (mean difference 1.42 [95% CI 0.91-1.92]; p<0.001), with fewer total complications (15 vs 37; p=0.003) and ureteric injuries (3 vs 9; p<0.001) in the simulation group. Although the number of procedures required to reach proficiency was similar, simulation-based training demonstrated higher overall proficiency scores than residents conventionally trained. Fewer procedures were required to achieve proficiency in the complex form of the index procedure with fewer serious complications overall.

***Clinical speciality keyword***

Surgery, Urology, Simulation

***References/Acknowledgements***

The authors are grateful to The Urology Foundation for funding this study.

***Ethics Statement***

The authors declare that all procedures followed were in accordance with the ethical standards of the responsible committee on human experimentation (institutional and national) and with the Helsinki Declaration of 1975 (In its most recently amended version).

Informed consent was obtained from all patients/participants included in the study.

#### 0012 Effect of Systems Simulation on Preparedness of Staff using a New Intraoperative MRI process

##### Kate Macfarlane^1^, Omair Malik^2^, Daniel Hufton^2^, Nathan Oliver^1^

###### ^1^Medical Education Directorate, NHS Lothian; ^2^Royal Hospital for Children and Young People

**Format:** Descriptive Work - Oral Presentations and Short Communications

**Topic:** New Technologies and Innovation

***Introduction***

Moving to a new hospital requires staff to engage with new equipment, care processes and communication methods. This can induce feelings of unpreparedness (1).

Intraoperative magnetic resonance imaging (ioMRI) had not previously been available for children undergoing neurosurgery in Edinburgh.

Systems integration simulation is emerging in the literature with a focus on testing new systems and processes, exposing potential latent safety threats before direct patient involvement. There is less focus on improving preparedness and the impact of simulation on this.

We explore how high-fidelity in-situ simulation can be used to allow teams and individuals to feel more prepared when using the ioMRI system in a new hospital.

***Description***

This evaluation aimed to explore the lived experience of 2 teams using a new ioMRI system, their sense of preparedness and how a simulation programme was helpful in addressing this.

The teams underwent a Patient Environment Simulation for Systems Integration (PESSI). The aim was to proactively test the process of ioMRI through high fidelity, psychologically safe simulation involving the teams and in-situ equipment working together in the new hospital.

The simulation faculty led a structured debrief. Key patient safety threats were reflected by the group and solutions discussed.

A questionnaire based on a 10-point Likert scale was distributed before and after completing the PESSI. The questionnaire was available through QR code link and paper copies. It evaluated different parameters of preparedness alongside qualitative feedback for faculty to reflect and learn from.

***Discussion***

16 multidisciplinary professionals completed the evaluation.

Staff average preparedness scores increased from 6.8 before the PESSI to 8.5 after completing the process. Knowing where to find materials and equipment improved from 6.7 to 7.6.

Perceived knowledge about obtaining medications improved from 6 to 7.9 and confidence with new communication systems improved from 5.1 to 7. When asked about preparedness to manage a real patient, staff average scores went from 6.9 to 7.8. Confidence in decision making also improved (7.4 to 8.1).

In line with social learning theory, many valued the video-link to observe the respective teams, share learning and collaborate best practice.

Based on feedback, the PESSI was adapted to include drills concentrating on fine-tuning sections staff felt most challenging. Fundamentally, realising that PESSI was useful for broad systems integration but fine-tuning drills were essential for staff preparedness.

Although effects on patient safety have not yet been evaluated, PESSI can be used in this and other new pathways to improve staff preparedness when transitioning hospitals prior to patient involvement.

***Clinical speciality keyword***

Paediatrics, Neurosurgery, Radiology

***References/Acknowledgements***

It Takes a Village to Move a Hospital: Simulation Improves Intensive Care Team Preparedness for a Move to a New Site Conall Francoeur, Sarah Shea, Margaret Ruddy, Patricia Fontela, Farhan Bhanji, Saleem Razack, Ronald Gottesman, Tanya Di Genova

Hospital Pediatrics Mar 2018, 8 (3) 148-156; DOI: 10.1542/hpeds.2017-0112

***Ethics Statement***

The authors declare that all procedures followed were in accordance with the ethical standards of the responsible committee on human experimentation (institutional and national) and with the Helsinki Declaration of 1975 (In its most recently amended version).

Informed consent was obtained from all patients/participants included in the evaluation.

This study was reviewed and is in concordance with NHS ethics advice.

#### 0013 Explaining health service preparation for the COVID-19 crisis utilizing simulation-based activities in a Norwegian hospital: A qualitative case study

##### Une Elisabeth Stømer^1^, Peter Dieckmann^2^, Thomas Laudal^2^, Kristi Bjørnes^2^, Sigrun Anna Qvindesland^1^, Hege Langli Ersdal^1^

###### ^1^Stavanger University Hospital; ^2^University of Stavanger

**Format:** Research Studies - Oral Presentations and Short Communications

**Topic:** Covid-19

***Introduction***

The first wave of the COVID-19 pandemic caused stress in healthcare all over the world (1-2). Hospitals and healthcare institutions had to reorganize their services to meet the demands of the crisis (3). The case hospital, a tertiary hospital in Norway, quickly initiated an upscaling of simulation- based activities to prepare the organization; while other hospitals chose to do the opposite and stop all simulation-based activities. The aim of this study is to explore the hospital leaders’ and simulation facilitators’ motivation and experiences of utilizing simulation-based activities in an unpredictable and stressful situation. We predefined three topics to explore:

1. What were the expectations of simulation-based activities in preparations for the pandemic?
2. What were the drivers and barriers for simulation-based activities during the pandemic?
3. How did simulation-based activities contribute during the first wave of COVID-19?

***Methods***

This is a qualitative case study utilizing semi-structured in-depth interviews. The data were analysed by thematic analysis according to Braun and Clarke (4).

***Results & Discussion***

Eleven hospital leaders and simulation facilitators were included in the study. We identified four themes explaining why COVID-19 related simulation-based activities were initiated and experienced consequences: 1. A multifaceted method including simulation faces a multifaceted crisis, 2. A well-established culture for simulation was crucial for scaling up simulation-based activities during the crisis, 3. Potential advantages outweighed potential risks for using simulation-based activities, and finally 4. Hospital leaders and simulation facilitators retrospectively assess the use of simulation-based activities as a feasible and appropriate way to prepare for a pandemic.

The hospital's decision to utilize simulation-based activities preparing for the COVID-19 crisis can be explained by many factors. First, it seems that many years of experience with systematic use of simulation within the hospital can explain the trust in simulation as a valuable tool. Second, both hospital leaders and simulation facilitators saw simulation as a unique tool for effective learning. Third, simulation-based activities fitted various challenges of the pandemic crisis.

Simulation-based activities provided crucial training and new competence but also revealed critical gaps in training and competence levels, treatment protocols, patient logistics, and environmental shortcomings, suggesting that organizational learning took place.

***Clinical speciality keyword***

N.A

***References/Acknowledgements***

**References:**

1. Ramsay S. Coronavirus: Italy's hardest-hit city wants you to see how COVID-19 is affecting its hospitals. Sky News; 2020 Available from:

https://news.sky.com/story/coronavirus-they-call-it-the-apocalypse-inside-italys-hardest-hit-hospital-11960597

2. Sharara-Chami, R., Sabouneh, R., Zeineddine, R., Banat, R., Fayad, J., & Lakissian, Z. (2020). In situ simulation: an essential tool for safe preparedness for the COVID-19 pandemic. Simulation in Healthcare, 15(5), 303-309.

3. Dieckmann, P., Torgeirsen, K., Qvindesland, S. A., Thomas, L., Bushell, V., & Ersdal, H. L. (2020). The use of simulation to prepare and improve responses to infectious disease outbreaks like COVID-19: practical tips and resources from Norway, Denmark, and the UK. Advances in Simulation, 5(1), 1-10.

4. Braun, V., & Clarke, V. (2006). Using thematic analysis in psychology. Qualitative research in psychology, 3(2), 77-101.

**Acknowledgements:**

The authors would like to thank the participants who generously shared their experiences form a demanding time.

***Ethics Statement***

The authors declare that all procedures followed were in accordance with the ethical standards of the responsible committee on human experimentation (institutional and national) and with the Helsinki Declaration of 1975 (In its most recently amended version). Informed consent was obtained from all the participants included in the study.

**Table 1 (abstract 0013).** Example of analytic process

Predefined topic	*Citation*	Sub-theme	Theme
**Driving factors for simulation- based activities**	*I think we had an advantage because our basic system (for using simulation) was in place. We did not need to establish a new basic system. (ID- 7)*	Long experience with simulation	A well-established culture for simulation was crucial for scaling up simulation activities during the crisis.
	*We are so integrated with simulation training at the XXX hospital that this (simulation) is what we use when something is difficult. We have even used simulation to learn how to avoid conflicts. It (simulation) is integrated as part of a toolbox that you can use for all sorts of weird things. (ID-8)*	Top-down anchoring of simulation/ managers see the benefit of simulation-based activities	

#### 0014 Exploring the experience of a multidisciplinary team with in-situ simulation training in an emergency non-operating room anesthesia procedure: a qualitative study

##### Caroline Guldberg Fugelli, Hege Langli Ersdal, Martin W. Kurz, Britt Sætre-Hansen

**Format:** Research Studies - Oral Presentations and Short Communications

**Topic:** Patient Safety / Quality Improvement

***Introduction***

Non-operating room anesthesia (NORA) care refers to administration of sedation/anesthesia outside the operating room to patients undergoing painful or uncomfortable procedures. These procedures are considered the most rapidly growing area in anesthetic patient load. Compared to anesthesia in the operating room, NORA represents new challenges to the anesthesia provider; e.g. less familiar procedures, room and equipment, patients with complex comorbidities and unknown team members.

In the Safer Stroke project, Stavanger University Hospital, regular multidisciplinary in-situ team-simulations for cerebral endovascular treatment of patients with acute large-vessel occlusion stroke was undertaken from 2017 to 2020.

The objective of this study was to explore how this regular simulation training in a low volume emergency NORA procedure, was experienced by team members, especially related to anesthesia care.

***Methods***

A qualitative exploratory study design was chosen, using focus group interviews. Study participants were selected through purposeful sampling, aiming for each focus group to be composed of one member from each profession in the endovascular treatment team.

Two of the authors carried out the interviews , using a semi-structured interview guide.

Content analysis by means of Granheim and Lundman constituted the method of data analysis and NVivo was used to analyze transcribed data from the interviews.

***Results & Discussion***

Preliminary analysis indicates that anesthesia providers are more uncomfortable and uncertain in NORA locations than in standard operation rooms.

Simulation training is experienced positive by the multidisciplinary team when it comes to assurance of procedural tasks, better team-work and communication, and understanding other professions responsibilities.

However, the existing simulation training has limitations, which contest some experienced main challenges in real life cerebral endovascular treatment procedures. Using a simulator, it was impossible to simulate an uncooperative sedated patient, which typically strain team communication in real situations. Team leaders are perceived to be less active and informative during clinical care, and there was little focus on anesthesia work and quality during the multi-professional simulations. A recurring problematic subject for the whole team was the neurologist as team leader. They were often perceived as too distressed to lead in an unfamiliar procedure and team environment.

Suggestions to improve simulation training in cerebral endovascular treatment were among others optimizing standardized team leader reports similar to “SafeSurgery” checklist, educate the team leader to provide common situational awareness, narrow and focus learning objectives, standardize to general anesthesia and introduce specific training challenges to anesthesia professionals.

***Clinical speciality keyword***

Anesthesia

***References/Acknowledgements***

1. Nagrebetsky A, Gabriel RA, Dutton RP, Urman RD. Growth of Nonoperating Room Anesthesia Care in the United States: A Contemporary Trends Analysis. Anesth Analg. 2017;124(4):1261-7.

2. Dabu-Bondoc S. NonOperating Room Anesthesia: distancing from invasive surgery, embracing the era of interventional medicine. Curr Opin Anaesthesiol. 2017;30(6):639-43.

3. Urman RD, Gross WL, Philip BK. Anesthesia Outside the Operating Room: Oxford University Press; 2018 2018-09.

4. Boggs SD, Barnett SR, Urman RD. The future of nonoperating room anesthesia in the 21st century: emphasis on quality and safety. Curr Opin Anaesthesiol. 2017;30(6):644-51.

5. Gendzel L, Bailey PD, Jr., Feldman JM. Not Them, Not Us, But We: The Importance of Teamwork in the NORA Environment. ASA Newsletter. 2013;77(11):12-4.

6. Salas E, Paige JT, Rosen MA. Creating new realities in healthcare: the status of simulation-based training as a patient safety improvement strategy. BMJ Qual Saf. 2013;22(6):449-52.

7. Graneheim UH, Lundman B. Qualitative content analysis in nursing research: concepts, procedures and measures to achieve trustworthiness. Nurse Educ Today. 2004;24(2):105-12.

8. Lindgren BM, Lundman B, Graneheim UH. Abstraction and interpretation during the qualitative content analysis process. Int J Nurs Stud. 2020;108:103632.

***Ethics Statement***

The authors declare that all procedures followed were in accordance with the ethical standards of the responsible committee on human experimentation (institutional and national ) and with the Helsinki Declaration of 1975 (In its most recently amended version).

Informed consent was obtained from all patients/participants included in the study.

All institutional and national guidelines for the care and use of laboratory animals were followed.

#### 0015 Faculty and students’ perceptions of High Fidelity Simulation: A study at an Emergency Medical Services school in King Saud University, Saudi Arabia

##### Majed Alqahtani

**Format:** Descriptive Work - Oral Presentations and Short Communications

**Topic:** Assessment using Simulation

***Introduction***

Background:

High Fidelity Simulation (HFS) can help the learner shift from knowledge to higher cognitive levels such as application and analysis (Zigmont et al., 2011). The goal of simulation is to provide experiential learning opportunities that allow the learner to apply theory to practice. Within the healthcare settings, it helps meet the needs of the growing, complex patient population and the shortage of clinical sites. Whilst a great deal of research has been conducted in medical and nursing education on the topic of High Fidelity Simulation, very little research exists that considers the experiences and needs of learners and teachers in Emergency Medical Services education (paramedics). In addition, a problem exists that teachers often feel ill-prepared to implement HFS as a teaching strategy and it has been acknowledged that faculty development to implement simulation is critical to an effective and sustainable simulation programme (Jeffries, 2014). Assessing the needs of both teachers and students regarding HFS implementation will aid in the development of interventions to address this issue.

***Description***

Aims:

The purpose of the study was to examine the views of faculty and students with respect to their experiences and challenges associated with the implementation of High Fidelity Simulation at King Saud EMS school. It was set to examine:

1. The extent to which faculty feel prepared to teach students in HFS settings?
2. How faculty prepare to teach in HFS settings?
3. What factors impact the implementation of HFS?
4. What are paramedic students’ and teachers perceptions of their experience of HFS?
5. What are paramedic students and faculty perceptions of the way in which HFS can be improved?

***Discussion***

Methodology:

A mixed methods approach was utilised, using interviews and surveys to tap into students and faculty perceptions.I used Simulation Design Scale (SDS) and Educational Best Practices (EPPS) surveys .The Interview were designed to tap into perceptions of preparedness and potential challenges.

Results:

The faculty and students agreed about having perceived the simulation design features and the educational practices elements to be present. ‘Statistical analysis of survey data suggest that students and faculty are equally satisfied with their experiences of HFS, as examined by the SDS and EPSS . It was the thematic analysis of the qualitative data that enabled the uncovering of challenges and barriers to HFS implementations. These challenges were categorised to themes associated with: institutional issues, support needs and assessment and feedback.

Conclusion

The findings will help inform the development of guidance and further support of educators in maximising this HFS as a teaching strategy to improve the students’ learning experience and preparation for their clinical role.

***Clinical speciality keyword***

paramedic, Emergency Medical Services

***References/Acknowledgements***

Hayden, J. K., Smiley, R. A., Alexander, M., Kardong-Edgren, S., & Jeffries, P. R. (2014). The NCSBN national simulation study: A longitudinal, randomized, controlled study replacing clinical hours with simulation in prelicensure nursing education. Journal of Nursing Regulation, 5(2), S3-S40.

Zigmont, J. J., Kappus, L. J., & Sudikoff, S. N. (2011, April). Theoretical foundations of learning through simulation. In Seminars in perinatology (Vol. 35, No. 2, pp. 47-51). WB Saunders.

***Ethics Statement***

The authors declare that they have followed the guidelines for scientific integrity and professional ethics. The article does not contain studies with human or animal subjects

#### 0016 Harnessing in situ simulation and organizational change: An ethnographic study of expansive learning in general practice

##### Gerry Gormley^1^, Richard Conn^1^, Anu Kajamaa^2^, Sarah O'Hare^1^

###### ^1^Queen’s University Belfast; ^2^University of Helsinki

**Format:** Research Studies - Oral Presentations and Short Communications

**Topic:** Interprofessional / Team Education

***Introduction***

Healthcare emergencies can be a matter of life or death. Interventions by healthcare professionals can have the barest of margins between benefit and harm. There are a range of healthcare contexts where such emergencies occur on a relatively infrequent basis, such as primary care. Nonetheless, the same degree of preparedness is required to optimise patient outcomes. Evidence is scant in how best to transform the state of preparedness in such environments. Insitu simulation offers opportunities to explore how best to enhance organizational change in the workplace. Change Lab (CL) (1) is a research assisted intervention, drawing upon Activity Theory (AT), that provides a framework to facilitate organizational change (expansive learning) (2, 3) (Figure 1). In this ethnographic observation study, we explored a microcycle of CL, using insitu simulation, and its impact in transforming the preparedness for managing an emergency event in a primary care setting.

***Methods***

A general practice and a range of healthcare workers were recruited for this study. A micro-cycle of CL was developed for the purpose of this study. Initially, an insitu simulation (i.e. a child presenting with Acute Anaphylaxis) was devised and conducted with participants. The simulation was video-recorded. Video footage was used as ‘mirroring data’ to allow participants to reflect on their response and identified challenges that hindered their performance. Participants were then asked to consider how to resolve these tensions and implement them into the workplace. A final insitu simulation was conducted to determine the enhancement of these implemented resolutions. Data was gained by focus groups, field notes and reflection diaries. A reflexive thematic analytical process was conducted, drawing upon AT, to discover the key elements of the activity systems, their interconnections, tensions and resolutions.

***Results & Discussion***

This micro-cycle of CL materialised organizational challenges that hindered optimal care of a critically ill patient. Collectively, participants were able to implement resolutions to many of these challenges into the workplace. The further insitu simulation confirmed that many of these resolutions transformed the preparedness in the practice. Moreover, a shift of culture took place in the practice and their continued willingness to engage in insitu simulation with the goal of improving patient care.

A microcycle of CL, harnessing insitu simulation, agentially transformed collective and organizational change in being the preparedness for managing a medical emergency in general practice. Moreover, such a model of expansive learning brought about a collective openness to agentially make real world changes to enhance their ability to provide patient care.

***Clinical speciality keyword***

In situ simulation, organisational change

***References/Acknowledgements***

1) Engeström Y, Virkkunen J, Helle M, Pihlaja J, Poikela R. The change laboratory as a tool for transforming work. Lifelong Learn Eur. 1996;1(2):10–7.

2) Engeström Y, Sannino A. Studies of expansive learning: foundations, findings and future challenges. Educ Res Rev. 2010;5(1):1–24. https://doi.org/10.1016/j.edurev.2009.12.002.

3) Engeström Y, Pyörälä E. Using activity theory to transform medical work and learning. Med Teach. 2021;43(1):7–13. https://doi.org/10.1080/0142159X.2020.1795105.

***Ethics Statement***

The authors declare that all procedures followed were in accordance with the ethical standards of the responsible committee on human experimentation (institutional and national ) and with the Helsinki Declaration of 1975 ( In its most recently amended version ).

Informed consent was obtained from all patients/participants included in the study.

All institutional and national guidelines for the care and use of laboratory animals were followed.

**Fig. 1 (abstract 0016).**
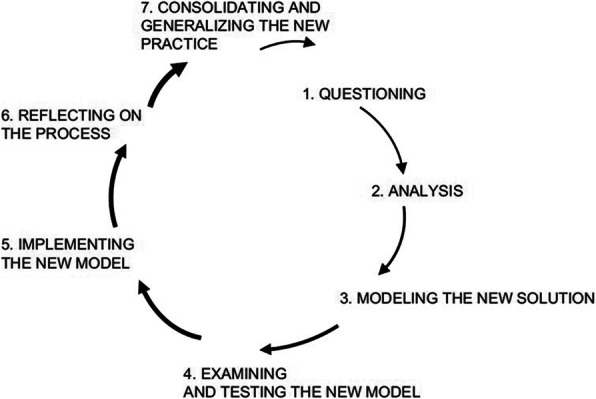
See text for description

#### 0017 How are teamwork skills applied in a multiplayer serious game by students and healthcare experts? A qualitative study

##### Mary Dankbaar, Tjitske Faber, Vicki Erasmus, Lara van Peppen

###### Erasmus University Medical Center

**Format:** Research Studies - Oral Presentations and Short Communications

**Topic:** Interprofessional / Team Education

***Introduction***

In healthcare, teamwork skills are critical for patient safety. Serious games may offer activating and efficient cognitive skills training, at a fraction of the costs of simulation settings (1). In our curriculum, interprofessional teamwork skills are trained using a blended design, starting with an e-module and online serious game (‘Team-Up!’). In Team-Up! players have to communicate (using one-to-one and team chats) in different roles in online simulated patient cases. After playing the game, students engage in face-to-face scenario training and a scenario assessment. A potential advantage of a blended design is a higher skills level at the start of face-to-face training, using expert resources more effectively (2).

***Methods***

In this qualitative study, our research question was what teamwork skills were used in the ‘Team Up!’ multiplayer game by medical students and teamwork experts. Findings can improve our understanding of the potential of serious games for training teamwork skills and can inform an effective blended training design on teamwork. 144 students and 24 healthcare experts in teamwork participated in our study, divided in groups of 4 players per scenario. We analyzed their one-to-one and team chats, using a deductive approach, with a conceptual framework based on Crew Resource Management (CRM) principles (3), including: Shared situational awareness, Decision making, Communication, Team management and Debriefing.

***Results & Discussion***

Results showed that most teamwork principles, e.g. shared situational awareness, decision making, communication, debriefing etc. were used in the game. Among students, these principles often were used on a more basic level, while specific aspects of CRM principles were missing, e.g. appointing a leader or prevention of fixation errors. Among the experts, we observed more specific principles, such as follow-up on actions or justification of decisions. However, some elements, such as prevention of fixation errors were also not observed. This may be caused by the somewhat artificial game environment (doing an online patient simulation, chatting instead of talking) or by the scenarios (some scenarios required less elaborate communication).

Conclusion: the Team-Up! game facilitates exercising teamwork skills on a basic level, with all important CRM principles. Online training of this skill seems to be limited by the artefacts of the game environment and scenarios. In the subsequent face-to-face training, more time can be spent on training more complex teamwork principles.

***Clinical speciality keyword***

Teamwork and CRM-based training

***References/Acknowledgements***

(1) Kalkman, C. J. (2012). Serious play in the virtual world: can we use games to train young doctors?. Journal of graduate medical education, 4, 11.

(2) Van Alten, D. C., Phielix, C., Janssen, J., & Kester, L. (2019). Effects of flipping the classroom on learning outcomes and satisfaction: A meta-analysis. Educational Research Review, 28, 100281.

(3) Gross, B., Rusin, L., Kiesewetter, J., Zottmann, J. M., Fischer, M. R., Prückner, S., & Zech, A. (2019). Crew resource management training in healthcare: a systematic review of intervention design, training conditions and evaluation. BMJ open, 9, e025247.

***Ethics Statement***

The authors declare that all procedures followed were in accordance with the ethical standards of the responsible committee on human experimentation (institutional and national ) and with the Helsinki Declaration of 1975 (In its most recently amended version).

Informed consent was obtained from all patients/participants included in the study.

**Fig. 1 (abstract 0017).**
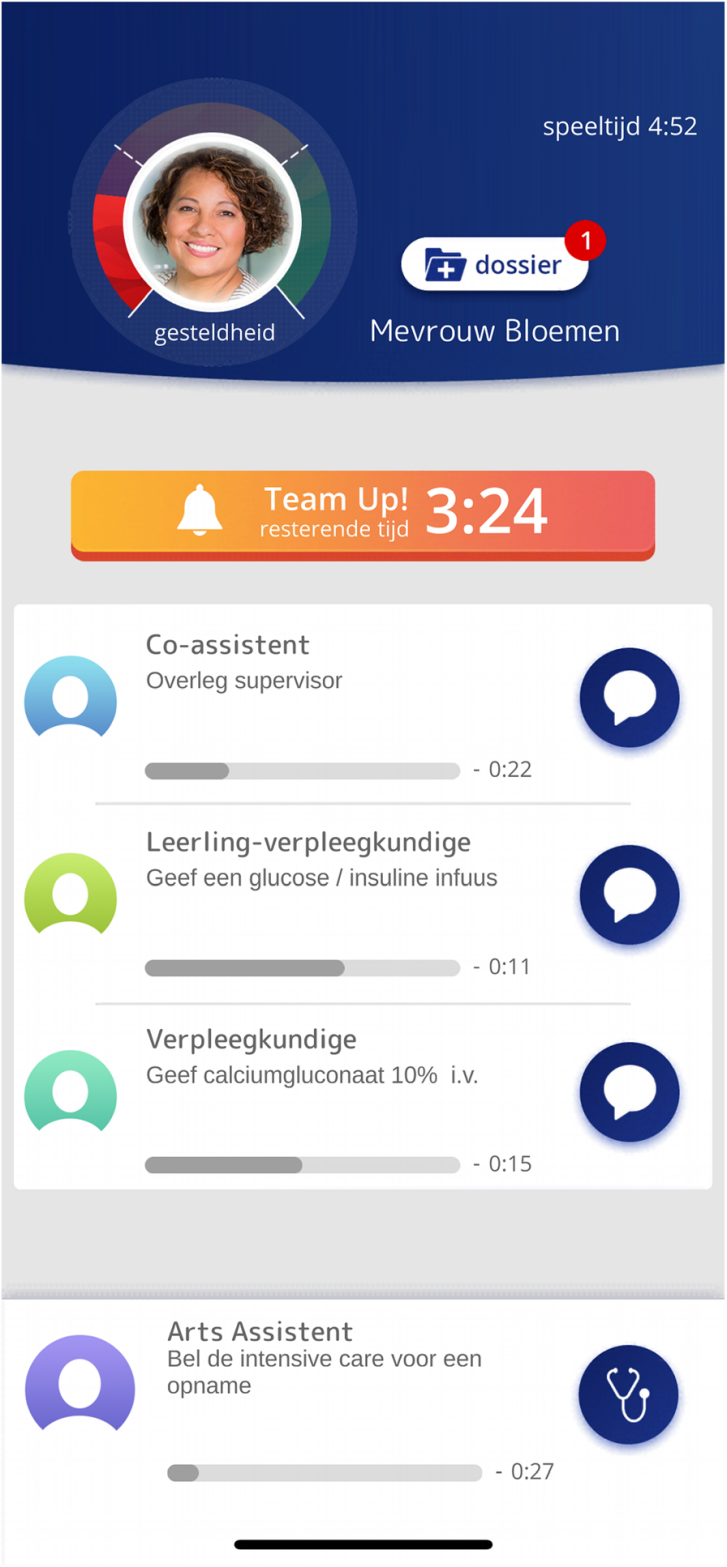
See text for description

#### 0018 Impact of stress on performance in a medical emergency simulation game

##### Tjitske Faber^1^, Mary Dankbaar^1^, Walter van den Broek^1^, Jeroen van Merriënboer^2^

###### ^1^Erasmus MC; ^2^Maastricht University

**Format:** Research Studies - Oral Presentations and Short Communications

**Topic:** New Technologies and Innovation

***Introduction***

Game-based learning (GBL) is gaining popularity in medical education, delivering engaging practice opportunities. Adding stressors to simulation training can either improve or impair learning and performance (1-3). Understanding how stress influences performance and learning in GBL can inform design choices. Specifically, objective or subjective stress responses may serve as indicators to guide adaptive scaffolding in the game. To improve insight into the relationships between stress and performance in GBL, our research questions are: (RQ1) does stress in GBL increase when creating a context using emotional content, social-evaluative content, and time pressure? and (RQ2) which measures of stress are valid indicators of performance in GBL?

***Methods***

Thirty medical students played three scenarios in a medical emergency game. For each scenario, a low, moderate or high-stress condition was created by reading a script with respectively low, moderate, or high amounts of emotional content, social-evaluative content, and time pressure. We measured subjective responses using the State-Trait Anxiety Index (STAI-DY1) and a cognitive appraisal index, calculated as the ratio between the perceived task demands and the learner’s perceived ability to deal with the task on a 1-7 Likert scale. A ratio > 1 (demands outweigh resources) implies a “threat” appraisal, while a ratio <1 implies a “challenge” appraisal. Physiologic responses (heart rate, heart rate variability (HRV) and galvanic skin conduction) were measured at baseline and throughout scenarios using the BioPac MP150 (Biopac Systems, Inc). We assessed performance using game score, systematicity in approach, and speed.

***Results & Discussion***

Regarding RQ1, we found no significant difference between conditions in subjective measures (STAI-DY1, pre-task cognitive appraisal, post-task cognitive appraisal) (resp. F = 0.043, p = .958; F = 0.432, p = .651; F = 0.995, p = .374, n=90). Preliminary results regarding physiological measures show no significant difference between conditions in HRV change from baseline (F = 0.285, p = .754, n=39). Regarding RQ2, preliminary results show a negative correlation between pre-task cognitive appraisal and score (rp = -.32, t = -3.2517, df = 88, p = .002). We will present further results at the conference.

Subjective stress did not differ between different stress conditions using scripts prior to gameplay. Conceivably, the engaging and immersive game limits the influence of the context. Pre-task cognitive appraisal correlated negatively with the game score, implying that students are well-calibrated in estimating their ability to successfully complete a scenario. This may be a promising indicator for adaptive support.

***Clinical speciality keyword***

emergency medicine

***References/Acknowledgements***

1. Morris CS et al. Motivational Effects of Adding Context Relevant Stress in PC-Based Game Training. Military Psychology. 2004;16(2):135-47.

2. Cumming SR, Harris LM. The impact of anxiety on the accuracy of diagnostic decision-making. Stress and Health. 2001;17(5):281-6.

3. LeBlanc VR et al. Paramedic Performance in Calculating Drug Dosages Following Stressful Scenarios in a Human Patient Simulator. Prehospital Emergency Care. 2005;9(4):439-44.

***Ethics Statement***

For studies using human or animal subjects:

The authors declare that all procedures followed were in accordance with the ethical standards of the responsible committee on human experimentation (institutional and national) and with the Helsinki Declaration of 1975 (In its most recently amended version). Ethical approval was waived by the local Ethical Committee.

Informed consent was obtained from all patients/participants included in the study.

#### 0019 Implementing an interprofessional simulation curriculum for undergraduate nursing and medical students: challenges and rewards

##### André Correia^1,2,3^, Manuela Castro^1,3,4^, Conceição Farinha^5^, Vasco Monteiro^1,3,6^, Marco Piedade^1,3,7^, Jorge Fonseca^1,3,8^, Helena José^5,9,10^, Alexandra Binnie^1,3,11^

###### ^1^Faculty of Medicine and Biomedical Sciences, Universidade do Algarve, Faro, Portugal; ^2^São João University Hospital Center, Porto, Portugal; ^3^Algarve Biomedical Center (ABC), Faro, Portugal; ^4^Balsa Family Health Unit, Tavira, Portugal; ^5^Universidade do Algarve, Faro, Portugal; ^6^National Institute of Medical Emergency, Portugal; ^7^University Hospital Centre of the Algarve, Faro, Portugal; ^8^Northern Devon Healthcare NHS Trust; ^9^Health Sciences Research Unit: Nursing (UICISA: E); ^10^European Academy of Nursing Science - EANS Scholar; ^11^William Osler Health System, Toronto, Ontario, Canada

**Format:** Descriptive Work - Oral Presentations and Short Communications

**Topic:** Interprofessional / Team Education

***Introduction***

Communication and collaboration play a key role in delivering safe healthcare to patients. These critical non-technical skills are common to both nursing and medicine, yet interprofessional education in these areas is not routinely integrated into the undergraduate nursing or medicine curriculum. Barriers to implementation include a lack of financial resources, a lack of faculty support, difficulty coordinating sessions between courses with different timetables, and a lack of perceived value.

***Description***

An interprofessional education program was designed to include all final-year medical and nursing students at the University of Algarve, in Faro, Portugal. Curriculum design and implementation was a collaborative effort between medical and nursing faculty members with experience in undergraduate education and simulation. The curriculum comprises two modules, each one incorporating theoretical and simulation sessions, with a focus on non-technical skills. In the first module, Teamwork, students are introduced to the principles of the TeamSTEPPS curriculum3. A 4-hour interactive workshop is followed by a 2-hour simulation session comprising 3 simulations scenarios in acute care medicine. Simulation debriefing focuses on teamwork performance framed within the TeamSTEPPS principles. In the second module, Patient Safety, the principles of patient safety are reviewed in a 4-hour interactive workshop followed by a 2-hour simulation session. Simulation scenarios incorporate patient safety issues as a prompt for discussion during simulation debriefing.

***Discussion***

To our knowledge, this is the first integrated interprofessional curriculum for nursing and medical students at the undergraduate level in Portugal. The program is now in its fourth year with more than 300 nursing and medical students having participated to date. Students who have completed the program rate it highly in terms of interest and practicality. They report improvements in their ability to communicate and to work in a team. They also report increased understanding of the challenges faced by students in the other course. Amongst medical students, over 90% feel that more interprofessional education should be incorporated into the medical curriculum.

In implementing this curriculum we faced several challenges. Among them were difficulties coordinating objectives and timing between two faculties; a lack of previous contact between medical and nursing instructors; a lack of previous collaboration between the two programs; and an imbalance in simulation experience between medical and nursing students. Since its implementation, reception of the interprofessional education program has been excellent on the part of both faculty and students, and has fostered greater connections between the two departments as well as opening the doors to future interprofessional educational collaborations.

***Clinical speciality keyword***

N.A

***References/Acknowledgements***

1. World Health Organization. ( 2010) . Framework for action on interprofessional education and collaborative practice. World Health Organization. https://apps.who.int/iris/handle/10665/70185

2. Homeyer et al. (2018). BMC nursing (17) 13.

3. Agency for Healthcare Research and Quality. (2006). TeamSTEPPS™ Guide to Action: Creating a Safety Net for your Healthcare Organization. AHRQ Publication No. 06-0020-4.

***Ethics Statement***

The authors declare that they have followed the guidelines for scientific integrity and professional ethics. The article does not contain any studies with human or animal subjects.

#### 0020 Impostor phenomenon in healthcare simulation educators

##### Kirsty Jane Freeman^1^, Stephen Houghton^2^, Sandra Carr^2^, Debra Nestel^3^

###### ^1^Duke NUS Medical School; ^2^The University of Western Australia; ^3^Monash University

**Format:** Research Studies - Oral Presentations and Short Communications

**Topic:** Faculty Development

***Introduction***

Have you ever experienced an overwhelming feeling of self-doubt, fearing that any minute now someone is going to discover you are a fraud and that you have been winging it the entire time? This is known as impostor phenomenon (IP) and is experienced along a continuum. At one end, the individual experiences occasional moment of self-doubt with minimal impact, however when experienced at the other end of the continuum where the feelings of impostorism are intense, IP has been linked to decreased job satisfaction, burnout, depression and interrupted career progression (1-3). A scoping review of IP in healthcare education revealed gaps in our understanding of the prevalence and influence of impostor phenomenon in healthcare educators (4). The aim of this study was to investigate the prevalence of IP in simulation educators (SE) and examine the effect of work-related characteristics on IP in the SE community.

***Methods***

148 SE from nine countries participated in an online survey. Along with questions related to demographic characteristics, IP was measured using two scales, the Clance Impostor Phenomenon Scale (CIPS) and the Leary Impostorism Scale (LIS). An exploratory factor analysis revealed that for both instruments a one-factor solution best fit the data, suggesting that all items in both measures fit onto a single theoretical construct. Both instruments demonstrated high internal reliability, with the Cronbach’s alpha for the CIPS being α = .96 and the LIS α = .95. Independent variables included gender, time spent on simulation activities per week, years working in simulation, and team size. This study was approved by the Human Ethics Research Office of The University of Western Australia (RA/4/20/5061).

***Results & Discussion***

Nearly half (46.6%) of the 148 SE who respondent to the survey experience frequent to intense feelings of impostorism (Table 1). A multivariate analysis of variance revealed no statistically significant interactions or main effects of gender, time spent on simulation activities per week, years working in simulation, and team size on impostor phenomenon.

This study shows that IP is prevalent across the healthcare SE community. The results suggest that feelings of impostorism do not decrease with more experience. Given the detrimental effects for both the individual and their employer, this presentation will discuss the implications for faculty development of the SE workforce.

***Clinical speciality keyword***

N/A

***References/Acknowledgements***

**References:**

1. Vergauwe J, Wille B, Feys M, De Fruyt F, Anseel F. Fear of being exposed: The trait-relatedness of the impostor phenomenon and its relevance in the work context. Journal of Business and Psychology. 2015;30(3):565-81.

2. McGregor LN, Gee DE, Posey KE. I feel like a fraud and it depresses me: the relation between the imposter phenomenon and depression. Social behavior and personality. 2008;36(1):43-8.

3. Villwock JA, Sobin LB, Koester LA, Harris TM. Impostor syndrome and burnout among American medical students: a pilot study. Int J Med Educ. 2016;7:364-9.

4. Freeman K, Carr S, Phillips B, Noya F, Nestel D. From clinician to educator: A scoping review of professional identity and the influence of impostor phenomenon. TAPS. in press;7(1).

5. Clance PR. The Impostor Phenomenon: when success makes you feel like a fake. Toronto: Bantam Books; 1985.

**Acknowledgements** - Ms. Pauline Clance for the permission to use the Clance Impostor Phenomenon Scale.

***Ethics Statement***

This study was approved by the Human Ethics Research Office of The University of Western Australia and the authors declare that all procedures followed were in accordance with their ethical standards. Informed consent was obtained from all participants included in the study.

**Table 1 (abstract 0020).**
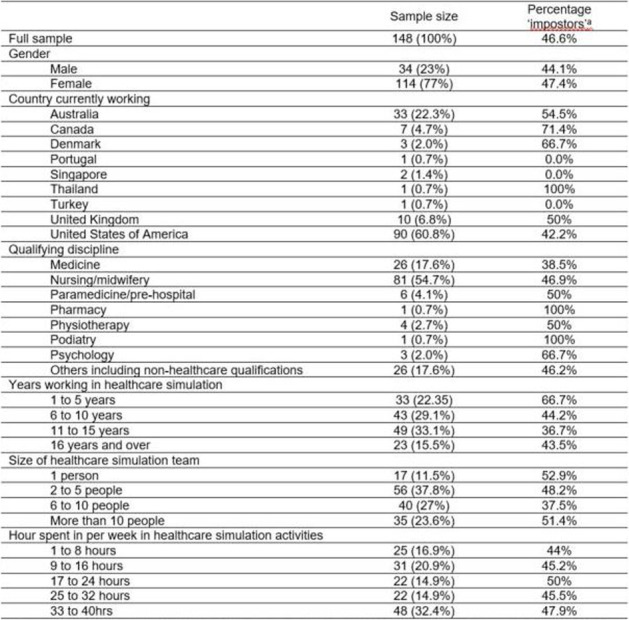
Demographic characteristics and percentage categorical impostors of the full sample (N=148) and within demographic categories of the sample

^a^ We used the cut-off score of 61 out of 100 to categorise SE as ‘impostors’ based on the scoring schema for the CIPS by Clance (5) where a score of 61 and above suggests frequent to intense IP experiences in the 20-item version of the CIPS

#### 0021 In-Situ On Call Shift (iSOCS): Using virtual “bulletin” boards to engender higher fidelity simulation

##### Gayatri Saxena, Alexander Kinsky

###### Ashford & St Peter's Hospitals NHS Foundation Trust

**Format:** Descriptive Work - Oral Presentations and Short Communications

**Topic:** New Technologies and Innovation

***Introduction***

Final year medical students continue to report feeling underprepared for independent working during “on-call” ward cover shifts (1,2), despite many universities implementing “Simulated-On-Call” programs, where students undertake tasks commensurate with typical shift requirements (3). One challenge for existing programs is student use of hospital software for the request and viewing of investigation results. Firstly, this requires student credentials for software containing patient-sensitive data. Secondly, creating artificial patients within such software is resource-intensive. Finally, short supply of ward-based IT hardware precludes student access without obstructing actual clinical duties.

This critical component of “on-call” working is therefore neglected in most programs, with no evidence of live information systems described in the literature (3–6).

The consequence is inferior program design with:

- Discrete tasks lacking adequate complexity (e.g. no task follow-up) (4,5)
- Unrealistic unidirectional information provision (4) (e.g. imaging under-utilised or results provided without request)
- Artificial environments (5,7) (e.g. Education Centres instead of wards)

We addressed these weaknesses in developing our in-Situ On Call Shift (iSOCS) program.

***Description***

The iSOCS program incorporated a broad range of tasks within a two-hour Medical Ward Cover shift. Importantly, simulated patient records and tasks were carried out in-situ in clinical environments except for one Simulation Suite activity. Students received pagers with instruction to triage and respond to bleeps appropriately. iSOCS concluded with “handover” and debrief with facilitators.

A novel, dynamic information sharing system was created using an online “bulletin” board (Padlet®). Students accessed online boards via smartphone or university-issued tablet, removing any need for student credentials for hospital software or the need for students to use coveted ward computers. Students viewed their individual board, initially containing only electronic radiology request forms. Facilitators then published investigation results on the board in real time, depending on student actions with realistic laboratory or imaging delays post-request.

***Discussion***

Video-recorded debriefs in conjunction with pre- and post-iSOCS questionnaires showed 30 student participants reporting increased confidence levels regarding medical on-calls. Students with experience of similar programs reported that iSOCS was “more useful” due to its in-situ nature and simulated electronic information system, which compelled more realistic engagement with tasks of appropriately high complexity. Students felt “quite stressed” during iSOCS, with comments remarking on the useful practical and psychological preparation for clinical practice. Current doctors piloted iSOCS and corroborated that it felt “almost exactly” like their on-call shifts.

Innovative use of existing technology, both inexpensive and easily replicable, unlocked the potential for high fidelity in-situ simulation.

***Clinical speciality keyword***

medicine

***References/Acknowledgements***

1. Van Hamel C, Jenner LE. Prepared for practice? A national survey of UK foundation doctors and their supervisors. Med Teach. 2015;37(2):181–8.

2. Monrouxe L V, Grundy L, Mann M, John Z, Panagoulas E, Bullock A, et al. How prepared are UK medical graduates for practice? A rapid review of the literature 2009–2014. BMJ Open. 2017;7(1):e013656–e013656.

3. Hawkins N, Younan HC, Fyfe M, Parekh R, McKeown A. Exploring why medical students still feel underprepared for clinical practice: a qualitative analysis of an authentic on call simulation. BMC Med Educ. 2021;21(1):1–11.

4. Wald D, Peet A, Cripe J, Kinloch M. A Simulated Night on Call Experience for Graduating Medical Students. MedEdPORTAL. 2016;1–7.

5. Kalet A, Zabar S, Szyld D, Yavner SD, Song H, Nick MW, et al. A simulated “Night-onCall” to assess and address the readiness-for-internship of transitioning medical students. Adv Simul. 2017;2(1):1–9.

6. Carpenter C, Keegan T, Vince G, Brewster L. Does simulation training in final year make new graduates feel more prepared for the realities of professional practice? BMJ Simul Technol Enhanc Learn. 2021;7(6):510–6.

7. Seale J, Ragbourne SC, Purkiss Bejarano N, Raj R, Whittingham L, Ikram S, et al. Training final year medical students in telephone communication and prioritization skills: An evaluation in the simulated environment. Med Teach [Internet]. 2019;41(9):1023–8. Available from: 10.1080/0142159X.2019.1610559

***Ethics Statement***

The authors declare that they have followed the guidelines for scientific integrity and professional ethics. The article does not contain any studies with human or animal subjects.

**Fig. 1 (abstract 0021).**
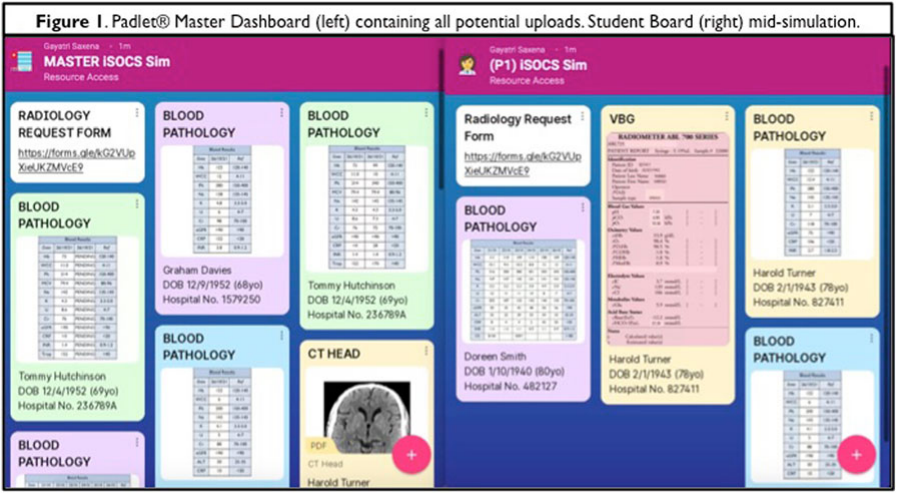
See text for description

#### 0022 In-situ stroke team training as tool for a bottom-up approach to quality assurance in hyperacute stroke care

##### Nele Elisa Bubel^1^, Johannes Sebastian Mutzenbach^1^, Slaven Pikija^1^, Cornelia Rösler^1^, Florian B. Lagler^2^

###### ^1^Department of Neurology, Christian Doppler Medical Center, Paracelsus Medical University, Salzburg, Austria; ^2^Institute for Inherited Metabolic Diseases and Department of Paediatrics, Paracelsus Medical University, Salzburg, Austria

**Format:** Descriptive Work - Oral Presentations and Short Communications

**Topic:** Patient Safety / Quality Improvement

***Introduction***

For patients with acute ischemic stroke, there are targeted, effective options for recanalization therapy as intravenous thrombolysis (IVT) and endovascular treatment (EVT). Recanalization therapy is primarily time-critical, as the effectiveness of treatment decreases dramatically over time. Process times as door to needle time (DNT) ware well established as markers for the quality of local stroke centres. Complex team interaction beyond disciplines is indispensable before starting any therapy: Ambulance personnel, emergency nurses, emergency physicians, stroke specialists, radiology technicians and neuroradiologists have to contribute to the process.

This constellation means a constant challenge for the interdisciplinary stroke team. Standard operating procedures serve as orientation for health professionals.

Simulation training has proven to be a way to improve individual skills as well as to practice and optimise workflow across different disciplines. Debriefing provides access to targeted re-sharpening of standard operating procedures (SOPs). Experiences with simulation training in emergency medicine have recently been introduced into acute stroke care. Application on local condition remains demanding.

***Description***

At our neurologic department, we plan to implement in-situ stroke team training. An in-situ setting is essential to derive practical insights to potential frictions in the workflow. A realistic stroke scenario is simulated from “door” to “needle”. Debriefing aims on team interaction, exactness of communication and human factors. Results of the debriefing are recorded and incorporated into the established processes. A quality circle, a board comprising of all involved disciplines, confirms changes in the protocol. The revised SOP is the basis for a repeated stroke team training. In this way, we create a bottom-up revision of SOP. Since it is a bottom-up method, we expect a better acceptance of the staff, as everyone has the opportunity to participate.

In this way, we aim to improve team interaction between the health professionals involved and ultimately reduce process times such as DNT. Consequently, the quality of care and patient safety will be increased.

***Discussion***

Simulation training for quality assurance is a recognised tool in emergency medicine. In stroke medicine, however, it is a young approach. As acute stroke care comprises more different disciplines than in usual emergencies, simulation training bears an even greater potential for quality improvement. In this context, quality improvement means faster application of IVT and performance of EVT, followed by better patient outcome after ischemic stroke. In-situ stroke team training provides a broad, open discussion of the existing SOP, a practice-oriented revision and finally a greater acceptance of changes within the medical staff.

***Clinical speciality keyword***

Neurology, Stroke medicine

***References/Acknowledgements***

Emberson J, Lees KR, Lyden P, Blackwell L, Albers G, Bluhmki E, u. a. Effect of treatment delay, age, and stroke severity on the effects of intravenous thrombolysis with alteplase for acute ischaemic stroke: a meta-analysis of individual patient data from randomised trials. The Lancet. November 2014;384(9958):1929–35.

Liebeskind DS, Jahan R, Nogueira RG, Jovin TG, Lutsep HL, Saver JL. Early arrival at the emergency department is associated with better collaterals, smaller established infarcts and better clinical outcomes with endovascular stroke therapy: SWIFT study. J NeuroInterventional Surg. Juni 2016;8(6):553–8.

Cook DA, Hatala R, Brydges R, Zendejas B, Szostek JH, Wang AT, u. a. Technology-Enhanced Simulation for Health Professions Education: A Systematic Review and Meta-analysis. JAMA. September 2011;306(9):11.

Tahtali D, Bohmann F, Rostek P, Misselwitz B, Reihs A, Heringer F, u. a. Crew-Ressource-Management und Simulatortraining in der akuten Schlaganfalltherapie. Nervenarzt. Dezember 2016;87(12):1322–31.

Bohmann FO, Tahtali D, Kurka N, Wagner M, You S-J, du Mesnil de Rochemont R, u. a. A Network-Wide Stroke Team Program Reduces Time to Treatment for Endovascular Stroke Therapy in a Regional Stroke-Network. Cerebrovasc Dis. 2018;45(3–4):141–8.

Ajmi SC, Advani R, Fjetland L, et al. Reducing door-to-needle times in stroke thrombolysis to 13 min through protocol revision and simulation training: a quality improvement project in a Norwegian stroke centre. BMJ Quality & Safety 2019;28:939-948.

***Ethics Statement***

The authors declare that they have followed the guidelines for scientific integrity and professional ethics. The article does not contain any studies with human or animal subjects.

**Fig. 1 (abstract 0022).**
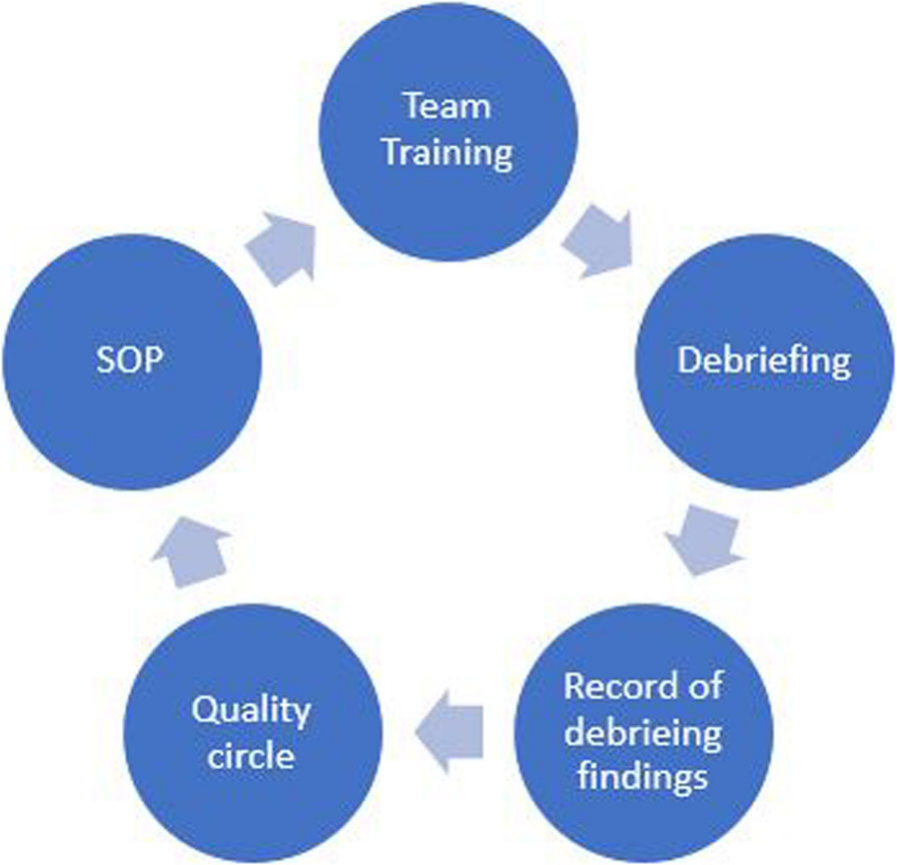
See text for description

#### 0023 Innovative Curriculum to Teach and Assess Basic Invasive Clinical Skills in Remote Settings

##### Dale Berg, Katherine Berg

###### Rector Clinical Skills and Simulation Center of Thomas Jefferson University

**Format:** Descriptive Work - Oral Presentations and Short Communications

**Topic:** Curriculum Development

***Introduction***

The Covid-19 pandemic prompted the need for remote education without compromising efficacy, student-faculty interaction, or deliberate practice in a simulated environment. This program was developed to provide on-demand, remote access to hands-on training modules for invasive procedural skills required for undergraduate and graduate medical education.

***Description***

The training modules consist of a fully-illustrated slide deck with instructional videos, equipment, and task trainers created by authors. Slide decks are designed reproducibly: background introducing the procedure, indications, contraindications, complications, teaching tips, an illustrated procedure checklist and annotated bibliography. Learners request a specific skills module and receive the instructional material along with a low-fidelity task-trainer for use in their home environment . After independently learning and practicing the procedure, learners are virtually coached and evaluated via the Zoom platform using Delphi-validated checklists created by Rector Clinical Skills and Simulation Center (RCSSC) faculty. This applies the method of skills attainment and development as described by Ericsson to a remote environment. Four procedure modules have been successfully piloted with senior medical students in the Rector Center's Advanced Physical Diagnosis course: male and female urinary bladder catheterization, orotracheal intubation, and nasogastric tube insertion. Seven student-physicians completed the pilot in their home environment; correct performance of checklist steps was 60% in pre-intervention, 86% in post-intervention assessment. Four additional kits have been developed: lumbar puncture, radial arterial line placement, intravenous catheter placement, and femoral central venous catheter placement. Remote learning is further expanded with a fully up-fitted van equipped as a mobile simulation classroom funded by the Measey Foundation. The students were allowed to determine the duration of deliberate practice hence tailoring the learning to the individual needs of the learner for that skill.

***Discussion***

All sessions were well received by students. The clinical environment is brought to the student, an innovation not only useful during the pandemic, but also in expanding advanced clinical education and deliberate practice to students and healthcare workers alike. By increasing access to technology-driven, high-quality curriculum and coaching, simulated clinical education can leave an impact beyond the classroom. This program is learner centered as the student determines need and length of time for deliberate practice. This program is reproducibly exportable; modules will be available for dissemination and complement standard task trainers used in clinical skills centers.

***Clinical speciality keyword***

Invasive clinical skills attainment and assessment; remote learning of skills

***References/Acknowledgements***

Berg K, Berg D, Riesenberg LA, Mealey K, Schaeffer A, Weber D, Justice EM, Davis J, Geffe K, Tinkoff G. The development of a validated checklist for Foley catheterization: preliminary results, Am J Med Qual. 2013.

Berg K, Berg D, Riesenberg LA, et al: The development of a validated checklist for nasogastric tube insertion: preliminary results. Am J Med Quality, 2013.

Ericsson, K. A. (2004). Deliberate practice and the acquisition and maintenance of expert performance in medicine and related domains. Academic Medicine, 10, S1–S12

Authors wish to acknowledge funding for this program by the “Benjamin and Mary Siddons Measey Foundation” of Philadelphia

***Ethics Statement***

The authors declare that they have followed the guidelines for scientific integrity and professional ethics. The article does not contain any studies with human or animal subjects.

#### 0024 Interprofessional clinical reasoning: A new team based immersive simulation for undergraduate students

##### Catriona Neil, Daniel Slack, Catherine Paton

###### NHS Lanarkshire

**Format:** Descriptive Work - Oral Presentations and Short Communications

**Topic:** Interprofessional / Team Education

***Introduction***

There is an increasing focus on clinical reasoning within all undergraduate health professions education. Clinical reasoning encompasses communication skills, using and interpreting diagnostic tests, understanding cognitive biases and human factors, critical thinking, patient centred evidenced based medicine and shared decision making. These skills happen individually and also within multidisciplinary teams (MDT). We were unable to find any literature on teaching clinical reasoning within the simulated environment. Given the known benefits of interprofessional education on both individuals and patient outcomes, bringing learners together in a simulated environment provides a safe space for them to develop these skills and reflect on their experiences.

***Description***

We developed an immersive interprofessional clinical reasoning simulation course, which takes place within a simulated acute medical receiving ward. The simulated patients have a variety of non-emergency issues, with specific clinical reasoning learning outcomes for each patient.

The team comprised a medical, nursing, physiotherapy and occupational therapy student and a pre-registration pharmacist, who worked together within the simulated environment. The learners received a handover and entered the ward for 30 minutes. The team decided how to prioritise tasks and deal with situations that arose. Two facilitators observed via the video/audio system. Following the simulation, the student’s handed back to the facilitators and participated in a 45-minute team systems based debrief. Twelve courses were run over 3 days, with 39 students attending in total. Feedback was collected through a questionnaire given to all students.

***Discussion***

The majority of students, 37/39, agreed or strongly agreed that they had found this a valuable learning experience. Within the feedback asking the most useful aspects of the experience, students commented on the realistic nature of the simulated environment including the pressures faced and ability to prioritise. They highlighted the value of working together, understanding others professional roles and the value of the debrief. They focused on being able to learn non-technical skills, communication, the transition from student to healthcare professional and more realistic MDT working.

This course fills a gap within the current curriculums, allowing interprofessional education to take place within a simulated clinical environment and thus learners practice their skills whilst understanding others roles. Learners developed their clinical reasoning skills within a safe environment, allowing them to continue their professional development. Following the results of this pilot, funding was awarded to repeat this course as a research project to gain a deeper understanding of the impact of the experience.

***Clinical speciality keyword***

N.A.

***References/Acknowledgements***

1. Cooper N, Frain J. Clinical Reasoning: An overview. In: Copper N, Frain J, editors. ABC of Clinical Reasoning. West Sussex: Wiley Blackwell; 2017. p. [1-5].

2. Reeves S, Fletcher S, Barr H, Birch I, Boet S, Davies N, McFadyen A, Rivera J, Kitto S. A BEME systematic review of the effects of interprofessional education: BEME Guide No. 39. Med Teach. 2016 Jul;38(7):656-68. doi: 10.3109/0142159X.2016.1173663. Epub 2016 May 5. PMID: 27146438

We would like to acknowledge the support of the National Skills Education Hub at NHS Louisa Jordan for their support in providing space to run this course and the input and support of Professor Jean Ker.

***Ethics Statement***

The authors declare that they have followed the guidelines for scientific integrity and professional ethics. The article does not contain any studies with human or animal subjects.

#### 0025 Post Incident Debrief- Team Talk

##### Anita Bignell, Sandra Parish, Emma Baxey, Marta Otega Vega, Kiran Virk, Gareth Evans

###### South London and Maudsley NHS foundation Trust

**Format:** Descriptive Work - Oral Presentations and Short Communications

**Topic:** Patient Safety / Quality Improvement

Introduction

The World Health Organization recommends clinical debriefing for team reflection after tasks, shifts or events1. Team debriefing is a key resource to improve team processes, enhance team effectiveness, bolster performance, and help organizations reflect and learn2. It supports shared reflective practice through collaborative learning and contributes to staff wellbeing and resilience by decreasing burnout3.

A multi-professional one-day course was developed with the aim of increasing the confidence of staff to conduct post-incident debriefs by introducing a selected evidence-based debrief model, the Team TALK framework. The framework was created to promote patient safety and a supportive culture of dialogue by guiding clinical teams to carry out short, structured and solution-based debriefings after everyday learning events4.

***Description***

The Post-Incident debrief course was delivered to a range of clinicians involved in working in inpatient settings including doctors, nurses, and allied health professionals. The learning objectives covered a range of skills such as increased knowledge in the role of debriefing in patient safety in mental health inpatient settings, as well as specific knowledge and techniques in using the Team TALK model to debrief colleagues. Additionally, the course had a focus on the importance of maintaining wellbeing at work and effectively identifying incidents related to work trauma.

Participants were introduced to the theory of the four distinct steps in the TALK framework. Then engaged in a series of interactive activities including watching content videos, breakout rooms and group discussions to practice, followed by guided reflective feedback from both facilitators and peers in a psychologically safe environment.

A pre and post survey was given to participants, asking them to rate their confidence in the learning objectives and self-efficacy using the Human Factors Skills for Healthcare Instrument. A paired samples t-test found significant improvements in confidence [t(19)=3.93, p<.001] and self-efficacy ratings [t(19)=4.35, p<.001], with an increase in total scores of 20.3 (18.5%) and 14.1 (11.8%) points respectively.

***Discussion***

The course was effective in helping staff identify clinical incidents that are convenient for debrief and increasing the knowledge and skills of participants in the use of the TEAM TALK framework for post-incident debriefs. Additionally, the course increased the confidence staff to identify the impact of trauma from incidents on both patients and staff, and the importance of maintaining the mental wellbeing of colleagues at the workplace to resultantly improve patients’ safety and care outcomes.

***Clinical speciality keyword***

Mental Health

***References/Acknowledgements***

1. Flin, J. Winter, C. Sarac, M.A. Raduma Tomas. Human Factors in Patient Safety: Review of Topics and Tools World Health Organization, Geneva (2009), p. 55

2. C.N. Lacerenza, S.L. Marlow, S.I. Tannenbaum, E. Salas. Team development interventions: evidence-based approaches for improving teamwork. Am. Psychol., 73 (4) (2018 May), pp. 517-531,

3. J. Chen, P.A. Bamberger, Y. Song, D.R. Vashdi. The effects of team reflexivity on psychological well-being in manufacturing teams. J. Appl. Psychol., 103 (4) (2018 Apr 1), pp. 443-462,

4. C. Diaz–Navarro, A. Hadfield, S. Pierce. TALK© Cardiff (UK); TALK Materials (2014)

***Ethics Statement***

Our organization has ethical approval from the King’s College London Psychiatry, Nursing and Midwifery Research Ethics Subcommittee to conduct research relating to the design and delivery of training (Reference: PNM1314-179).”

#### 0026 Preparing Simulated Participants for Feedback Practices in Communication Skills Training

##### Clare Sullivan^1^, Andre Doyle^1^, Naoise Collins^1^, Michelle O'Toole^1^, Claire Mulhall^1^, Nancy McNaughton^2^, Walter Eppich^1^

###### ^1^RCSI University of Medicine and Health Sciences; ^2^The Michener Institute of Education at UHN

**Format:** Research Studies - Oral Presentations and Short Communications

**Topic:** Faculty Development

***Introduction***

Despite increasing evidence that demonstrates the value of feedback practices, we are only beginning to understand how these practices influence learning through simulated participant (SP) methodology1. Although feedback practices comprise a key contribution to student learning (2), SPs report that they find the delivery of precise and inspiring feedback difficult (3). Suboptimal feedback practices may impede learning. SPs are recruited from different groups, including; professionally trained actors, volunteer or paid lay people and health professions educators. These individuals come to their role with different experiences, knowledge and training due to their diverse backgrounds. These backgrounds give them specific strengths and weakness in their simulated role portrayal related to cost, training needs, feedback quality and simulation fidelity (6). The aim of this study is to explore how SPs from different groups prepare for and engage in feedback practices for communication skills training.

***Methods***

Using grounded theory methodology (4), we are conducting a qualitative study with SPs who have participated in a simulated role (patient, family member or an embedded role as healthcare worker) for communication skills training. The RCSI research ethics committee approved the study, REC 202103009. Through iterative data collection and analysis, we are conducting semi-structured interviews with SPs. Participants have been recruited from three different educational bodies and across the three groups (faculty, professional actors and lay SPs).

***Results & Discussion***

Preliminary results indicate that in order to prepare for simulated roles and feedback, SPs participate in training, prepare from the scenario script and draw on their own experiences. For new roles they sometimes feel unprepared, motivating them to seek out further guidance from faculty or colleagues. During role portrayal SPs benefit from understanding the learning objectives as this helps them scaffold the learning towards the desired objectives. After the scenario SPs foster an environment which is psychologically safe for open discussion, they encourage student reflection and they help students to understand the patient’s perspective. SPs learn from their experiences portraying roles and this feeds into their future roles. It is important for educators to understand the training needs of SPs. SPs have a desire to perform well and continually improve. Understanding how to provide better supports to SPs to engage in feedback practices has the potential to improve the learning experience for students. These insights will inform future SP selection and role preparation.

***Clinical speciality keyword***

N.A.

***References/Acknowledgements***

1. BOKKEN, L., LINSSEN, T., SCHERPBIER, A., VAN DER VLEUTEN, C. & RETHANS, J. J. 2009. Feedback by simulated patients in undergraduate medical education: a systematic review of the literature. Med Educ, 43, 202-10.

2. MCLEAN, M., JOHNSON, P., SARGEANT, S. & GREEN, P. 2015. Simulated patients’ perspectives of and perceived role in medical students’ professional identity development. Simul Healthc, 10, 85-91.

3. NESTEL, D., CLARK, S., TABAK, D., ASHWELL, V., MUIR, E., PARASKEVAS, P. & HIGHAM, J. 2010. Defining responsibilities of simulated patients in medical education. Simul Healthc, 5, 161-8.

4. NESTEL, D., CLARK, S., TABAK, D., ASHWELL, V., MUIR, E., PARASKEVAS, P. & HIGHAM, J. 2010. Defining responsibilities of simulated patients in medical education. Simul Healthc, 5, 161-8.

5. CHARMAZ, K. 2014. Constructing Grounded Theory, SAGE.

6. MAVIS, B., TURNER, J., LOVELL, K. & WAGNER, D. 2006. DEVELOPMENTS: Faculty, Students, and Actors 212 as Standardized Patients: Expanding Opportunities for Performance Assessment. Teaching and 213 Learning in Medicine, 18, 6.

***Ethics Statement***

The study was approved by the RCSI Research ethics committee reference REC 202103009. Informed consent was obtained from all participants included in the study.

#### 0027 Psychometric properties of the Norwegian Version of The Clinical Learning Environment Comparison Survey

##### Camilla Olaussen, Lars-Petter Jelssness –Jørgensen, Christine Raaen Tvedt, Dag Hofoss, Simen Alexander Steindal

**Format:** Research Studies - Oral Presentations and Short Communications

**Topic:** Assessment using Simulation

***Introduction***

This study was driven by the increasing use of patient simulation in Norwegian nursing education, and the need for valid tools to guide educators in their work to develop simulation experiences that may compensate for learning needs in the clinical environment. To evaluate the clinical and simulated practice so that both strategies can be optimally combined in nursing education programmes, valid tools are needed. The aim of this study was to translate the Clinical Learning Environment Comparison Survey (CLECS) into Norwegian and to evaluate its psychometric properties.

***Methods***

The CLECS was translated into Norwegian following the World Health Organization guidelines, including forward-translation, expert panel, back-translation, pre-testing and cognitive interviewing. Nursing students at a Norwegian university college participated in the study (psychometrical testing) based on informed consent. Reliability and validity of the translated version of CLECS was investigated using a confirmatory factor analysis (CFA), Cronbach’s alphas, and test-retest analysis.

***Results & Discussion***

A total of 122 nursing students completed the questionnaire, and Cronbach alphas for the CLECS subscales ranged from .69 to .89. CFA goodness-of-fit indices (χ2/df. = 1.409, CFI = .915, RMSEA = .058, pclose = .15) showed acceptable model fit. Test-retest ICC ranged from .55 to .75, except for two subscales with values below .5.

The CLECS (Norwegian version) showed acceptable construct validity and the Cronbach alpha values for all hypothesized subscales demonstrated internal consistency. The majority of subscales displayed moderate to good test-retest reliability. However, in two subscales there were observed problematic reliability. One goodness of fit indicator that speaks against the fit of the model is the χ2’s p-value of less than .001. However, the χ2-df ratio was well below the recommended limits by Byrne (1989) and the pclose and the RMSEA both met the criteria suggested by Browne and Cudeck (1993). An important step in improving nursing students’ clinical education is to understand how learning needs are met by the two methods of learning. Until now, no valid instrument that provide educators the direction on how to ensure an optimal combination of clinical and simulated experiences has been available in Norway. The CLECS could be integrated in Norwegian nursing education for course and program evaluations. CLECS findings may also be used to guide nursing educators in their work to develop and refine simulation experiences that may compensate for students learning challenges in clinical practice.

***Clinical speciality keyword***

N:A

***References/Acknowledgements***

World Health Organization. (2018). Process of translation and adaption of instruments. Retrieved from http://www.who.int/substance_abuse/research_tools/translation/en/

Byrne, B. M. (1989). Basic applications and programming for confirmatory factor analysis models. New York: Springer-Verlag.

Browne, M., & Cudeck, R. (1993). Alternative ways of assessing model fit. In K. A.

Bollen & J. S. Long (Eds.), Testing structural equation models (pp. 136-162). Newbury Park, California: Sage.

***Ethics Statement***

The authors declare that they have followed the guidelines for scientific integrity and professional ethics. The article does not contain any studies with human or animal subjects.

#### 0028 Remote Virtual sim- Debriefing pre-filmed 360 videos of simulations over a video-conferencing platform

##### Tim Mason^1^, Nick Peres^2^

###### ^1^North Devon healthcare NHS trust; ^2^Torbay Hospital/ HEE

**Format:** Descriptive Work - Oral Presentations and Short Communications

**Topic:** New Technologies and Innovation

***Introduction***

Simulation is resource heavy in terms of time, space, equipment, and faculty, with limitations on the number of people you can deliver sessions to. During the COVID-19 pandemic, social distancing makes face to face simulation more challenging.

After working with a local healthcare VR team through a return to training project¹, we explored benefits and acceptability of remotely debriefing 360° videos of simulated paediatric emergencies. Remote debrief has been used successfully to train teams2 and faculty3. However, it’s use in the context of 360 content is still novel. The theory being that because the participant is an “active observer” this would bring a more immersive experience.

We were keen to assess whether this method of experiencing simulation teaching was engaging, immersive and safe.

***Description***

Using 360° content filmed at RD&E hospital, we ran a remote virtual simulation sessions over MS Teams video conferencing platform.

After a quick pre-brief we asked the learners to watch a pre-filmed scenario (https://youtu.be/KpQl0VUfERI). We guided them to interact with the video, directing their view in the 360° media to whatever interested them. This was followed by a debrief using a modified “standard” simulation debrief structure.

From April 2020 to Nov 2021. We ran 15 Sessions with over 150 learners, 87 provided feedback (unable to get formal feedback from regional/ National courses)

Used for local/regional/national teaching. Group sizes ranged from 4 to 40 learners.

***Discussion***

Feedback was globally positive, 100% ‘would do it again’. Qualitative feedback was rich and promising:

“more life-like than expected”

“safer than real sim as I didn’t feel judged”

“360 element meant you had a different perspective”.

Debrief often led to learners sharing stories of clinical encounters and it did not matter that they had not actively participated in the simulation.

Additional positives include:

- Easy accessibility for anyone to view 360 video.
- No additional faculty or manikins.
- Relatively quick and engaging to run remotely.
- Larger groups can be engaged than standard simulation.
- Immersive experiences can be repeated.

After presenting this work in 2020, other hospitals have taken our pre-made scenarios we have used this as part the national RCPCH Sepsis courses in May and October 21.

We recommend remote 360 sim with debrief as a fantastic adjunct to standard simulation. The modality is low cost, well received and a safe method of providing people with an experience of emergency scenarios. This is enhanced though a supportive debrief.

***Clinical speciality keyword***

Paediatrics

***References/Acknowledgements***

1. Mason T G627(P) Is virtual resus training the future? Can 360 degree ‘immersive video’ engage paediatric trainees safely in life support training? – A project as part of the south west return to paediatric training course (SWRPT) Archives of Disease in Childhood 2019;104:A254.

2. Meaklim, A.. (2016). Remote debriefing - A new paradigm for low resource and rural hospitals?. 31. 59-62.

3. Gross IT, Whitfill T, Auzina L, et al Telementoring for remote simulation instructor training and faculty development using telesimulation BMJ Simulation and Technology Enhanced Learning Published Online First: 18 May 2020. doi: 10.1136/bmjstel-2019-000512

***Ethics Statement***

The authors declare that they have followed the guidelines for scientific integrity and professional ethics. The article does not contain any studies with human or animal subjects.

#### 0029 SimBegin - A novel simplified Train The Trainer-program and a potential gamechanger for dissemination of simulation-based training

##### Kjetil Torgeirsen^1^, Patrik Nyström^2^, Paschal Mdoe^3^, Estomih Mduma^3^, Benjamin Kamala^3^, Asmita Acharya^4^, Rashmi GV Aradhya^4^, Hege Ersdal^5^

###### ^1^SAFER and Faculty of Health Sciences, University of Stavanger; ^2^SAFER, Safety Factors Finland; ^3^Haydom Lutheran Hospital; ^4^Laerdal Medical India; ^5^Department of Anaesthesia, Stavanger University Hospital and Faculty of Health Sciences, University of Stavanger

**Format:** Descriptive Work - Oral Presentations and Short Communications

**Topic:** Faculty Development

***Introduction***

SimBegin is a Train The Trainer-program developed as a collaboration between SAFER simulation center and Laerdal Medical aiming to address the skills of briefing a scenario, run a scripted scenario, and performing a debriefing. The content is designed as an entry level for beginners in simulation and debriefing.

SimBegin development was guided with the idea of scalability in all settings including low resource countries. Development took place during the pandemic which necessitated the development team to search new ideas and solutions, in a world where traditional physical delivery methods and travel were not possible

***Description***

The initial course utilizes e-learning, webinars, and face to face training. Materials are presented in text, pictures, animations, videos, and short assessment questionnaires. Participants are working both individually with the e-learning, in pairs and in groups during the workshops. All activities are guided and facilitated by experienced simulation facilitators.

In addition to the initial course, the SimBegin program offers an optional follow-up, including mentoring and a faculty development process. The program aims to establish local faculties enabling sustainable systems for further dissemination and scale up of simulation based training. Description of the initial course, content of modules and how this course develops into a program, can be found in Fig.1 (SimBegin program 3 step structure).

***Discussion***

We believe that SimBegin is a potential gamechanger for dissemination of simulation-based training. The simplification made by removal of complex topics from the entry level course, allows the participant to focus more on the structure in the debriefings. The simplification also makes the faculty development process easier and faster.

The full scale up of the SimBegin program is ongoing in 5 regions in Tanzania, including 30 health care facilities as an integrated part of the Safer Births Bundle of Care project funded by the Global Financing Facility. This project includes research initiatives investigating the outcomes of the different but bundled interventions.

Based on observations and experiences from India, Nepal, Tanzania, Ethiopia, DR Congo and faculty development processes ongoing in India and Tanzania, the SimBegin program is expected to: 1. Help participants to execute structured, functional and reflection driven debriefings. 2. Help participants reaching the level of facilitator-skills that would be expected of any train the trainer programs. 3. Be quickly scalable. 4. Be possible to run virtually, with certain requirements.

***Clinical speciality keyword***

N.A

***References/Acknowledgements***

SimBegin was developed as a collaboration between SAFER and Laerdal Medical, funded by Innovation Norway through the Visjon 2030 program.

The SimBegin development team; Patrik Nyström (SAFER/Laerdal consultant), Kjetil Torgeirsen (SAFER), Unni Silkoset (Laerdal Medical), Rashmi GV Aradhya (Laerdal Medical India) Hilde Hetland (SAFER), Christian Søreide (SAFER), Elsa Søyland (SAFER).

***Ethics Statement***

The authors declare that they have followed the guidelines for scientific integrity and professional ethics. The article does not contain any studies with human or animal subjects.

**Fig. 1 (abstract 0029).**
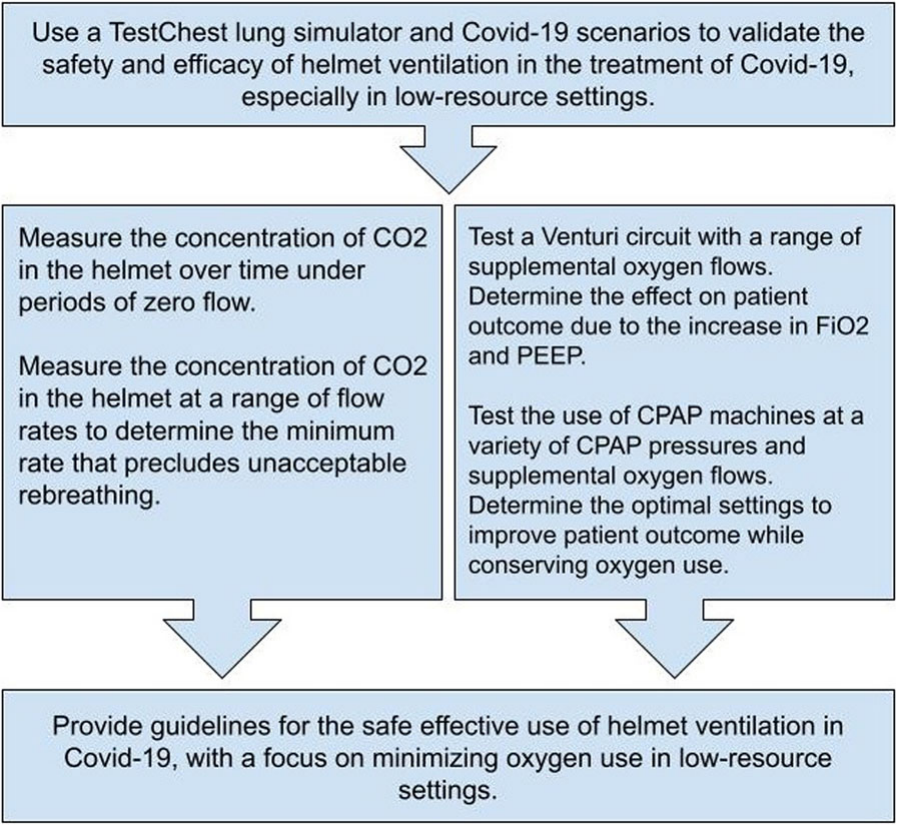
See text for description

#### 0030 Simulation Based Mastery Learning for Viral Haemorrhagic Fever Personal Protective Equipment Donning and Doffing Training

##### Callum Peter Mutch, Nathan Oliver, Vicky Tallentire, James Tiernan, Bozena Poller

###### NHS Lothian

**Format:** Descriptive Work - Oral Presentations and Short Communications

**Topic:** Assessment using Simulation

***Introduction***

Pandemic preparedness is a key priority for healthcare services and governments following the COVID-19 pandemic. High Consequence Infectious Diseases (HCIDs), including Viral Haemorrhagic Fevers (VHF), have the potential to cause pandemics and require particular focus for preparedness due to their high mortality rates. Personal Protective Equipment (PPE) for HCIDs is more complex and challenging to use safely than standard droplet or aerosol protection PPE, is associated with Health Care Worker (HCW) anxiety, and carries significant risk of HCW contamination if done incorrectly. Developing an efficient and effective method to both train and assess HCWs in the skills of Donning and Doffing HCID PPE is an important facet of preparedness.

***Description***

Simulation Based Mastery Learning (SBML) is an established methodology for skills training (1). Previous work has explored SBML for training of PPE use for COVID-19 (2,3). A recent study has explored the use of SBML enhanced with an asynchronous pre-learning package to maximize the efficiency of synchronous learning time (4).

Learners are provided an interactive pre-learning package with exemplar videos of the donning and doffing process. Following this, synchronous learning takes place with faculty facilitating two learners who undergo formative assessment with feedback followed by a summative assessment. Questionnaires are completed by learners at 4 stages: prior to pre-learning, following pre-learning, following assessment session and at 3 months following assessment session.

***Discussion***

HCID are a rare but important clinical presentation to Emergency Departments and Infectious Diseases Units. Given the infrequency of clinical encounters it is important to ensure that HCWs are adequately trained and assessed to be safe in their donning and doffing procedures with ongoing refresher training to maintain competency. The required frequency of this training is poorly understood and most studies do not undertake longer term follow up of training outcomes. The majority of literature on the use of simulation training for HCID PPE is related to immersive simulation (5), however this can be resource and faculty intensive and a SBML approach is better suited to assessment of competency in a process where inter-user variance should be minimized. By surveying participants following their learning session we can explore how their self-assessed preparedness changes over time and how this is influenced by their clinical activity in this time period.

***Clinical speciality keyword***

Infectious Diseases

***References/Acknowledgements*** Acknowledgements:

Dr Oliver Koch, Consultant Physician, Regional Department of Infectious Diseases, NHS Lothian Michelle Wiseman, Senior Charge Nurse, Regional Department of Infectious Diseases, NHS Lothian

References:

1. McGaghie WC, Harris IB. Learning Theory Foundations of Simulation-Based Mastery Learning. Simul Healthc. 2018;13(3S Suppl 1):S15–20.

2. Doverty J, Anderson R, Seglah O. PP19 The use of simulation based mastery learning for donning and doffing personal protective equipment: effective training for multidisciplinary health care workers. BMJ Simul Technol Enhanc Learn [Internet]. 2020 Nov 1 [cited 2021 Nov 15];6(Suppl 1):A23.2-A24. Available from: https://stel.bmj.com/content/6/Suppl_1/A23.2

3. Pokrajac N, Schertzer K, Poffenberger CM, Alvarez A, Marin-Nevarez P, Winstead-Derlega C, et al. Mastery Learning Ensures Correct Personal Protective Equipment Use in Simulated Clinical Encounters of COVID-19. West J Emerg Med [Internet]. 2020;21(5):1089–94. Available from: http://ovidsp.ovid.com/ovidweb.cgi?T=JS&PAGE=reference&D=med17&NEWS=N&AN=32970559

4. Scahill EL, Oliver NG, Tallentire VR, Edgar S, Tiernan JF. An enhanced approach to simulation-based mastery learning: optimising the educational impact of a novel, National Postgraduate Medical Boot Camp. Adv Simul. 2021;6(1):1–10.

5. Verbeek JH, Rajamaki B, Ijaz S, Sauni R, Toomey E, Blackwood B, et al. Personal protective equipment for preventing highly infectious diseases due to exposureto contaminated body fluids in healthcare staff. Cochrane Database Syst Rev [Internet]. 2020 Apr 15 [cited 2021 Nov 15];2020(4). Available from: /pmc/articles/PMC7158881/

***Ethics Statement***

The authors declare that they have followed the guidelines for scientific integrity and professional ethics. The article does not contain any studies with human or animal subjects.

**Fig. 1 (abstract 0030).**
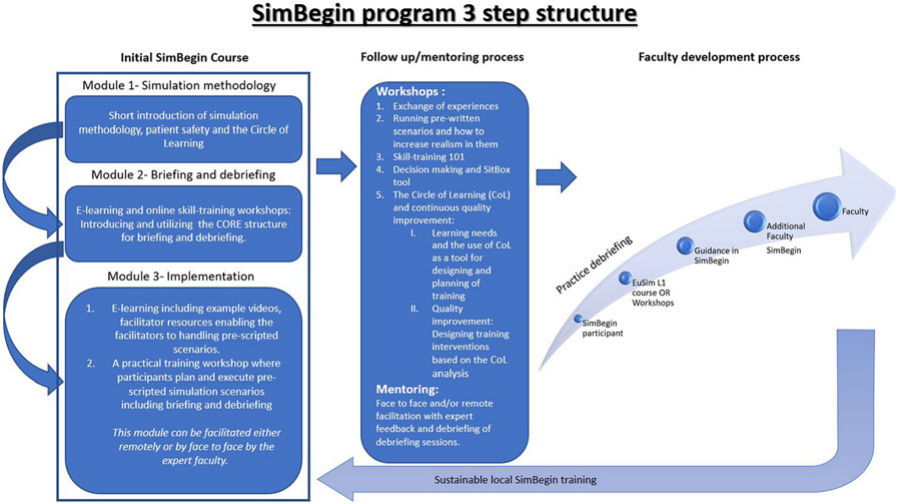
See text for description

#### 0031 SIMULATION HELPS ANESTHESIOLOGIST RESIDENCY TRAINING DURING THE PANDEMIC

##### Mariana Luís, Regina Rodrigues, Sara Freitas

###### Madeira Clinical Simulation Center, Regional Health Service of the Autonomous Region of Madeira (SESARAM)

**Format:** Descriptive Work - Oral Presentations and Short Communications

**Topic:** Covid-19

***Introduction***

The COVID-19 pandemic, by limiting the movement and number of health professionals between hospitals and in the operating room, created greater difficulties for anesthesiology residency training, especially in isolated regions dependent on airport connections. Believing that clinical simulation can minimize this adversity, the authors present the Simulation Training Plan put into practice at our hospital during the pandemic, which aimed to help the training of seven anesthesiology residents in acquiring and certifying skills.

***Description***

This Simulation Training Plan aims to allow the practice of rare and complex situations, with low incidence and great impact for the patient and the anesthesiologist.

The Plan had a total duration of 24 hours (21 of practical training and 3 of theoretical exposition), divided into 6 modules of 4 hours each, and was aimed at 7 trainees, with a ratio facilitator/trainee of 1:2. The facilitators were certified EuSIM instructors.

Each module explored 2 action algorithms for critical situations, using high-fidelity mannequins, in a training room environment of the Simulation Centre, except the last module, which was carried out in a realistic environment of an operating room, involving doctors, nurses and operational assistants. In total, 12 major algorithms from the anesthesiology curricula were trained. The airway approach in the COVID-19 patient, the training of non-technical skills and the Crisis Resources Management (CRM) methodology were present throughout the training. Great importance was given to debriefing as a learning tool. Hygienic safety rules were fully complied.

An anonymous satisfaction questionnaire was applied to all 7 trainees, 6 months after the last module. A modified version of the Satisfaction with Simulation Experience (SSE) Scale was used, considering only 9 items related with clinical reasoning and clinical learning. The answers are shown in table 1. All participants strongly agreed or agreed with the benefits of simulation, none opposed

***Discussion***

With this Training Plan, we hope to have improved the skills and confidence of anesthesiology residents, increasing patient and healthcare professional safety, as well as the outcomes of COVID and non-COVID patients during the pandemic and post-pandemic period.

It also facilitates communication between residents and experienced anesthesiologists in a calm environment, allowing high quality knowledge acquisition and team building.

Bearing in mind the manifested satisfaction with the Simulation Training Plan, the authors recommend that all six modules are seen as mandatory during residency.

***Clinical speciality keyword***

Anesthesiology training

***References/Acknowledgements***

1. Diário da República, 1.ª série — N.º 74 — 15 de abril de 2016, Portaria n.º 92-A/2016 de 15 de abril

2. Anesthesiology Studies Program Specific training, 2017, Doctor's Order

https://ordemdosmedicos.pt/wp-content/uploads/2017/09/Programa-de-Estudos-em-Anestesiologia_vsfinal2_27Set17.pdf

3. Gilligan, C., Lapkin, S., & Jones, D. (2011). The development and psychometric testing of the Satisfaction with Simulation Experience Scale. Nurse Education Today.

***Ethics Statement***

The authors declare that they have followed the guidelines for scientific integrity and professional ethics. The article does not contain any studies with human or animal subjects.

**Fig. 1 (abstract 0031).**
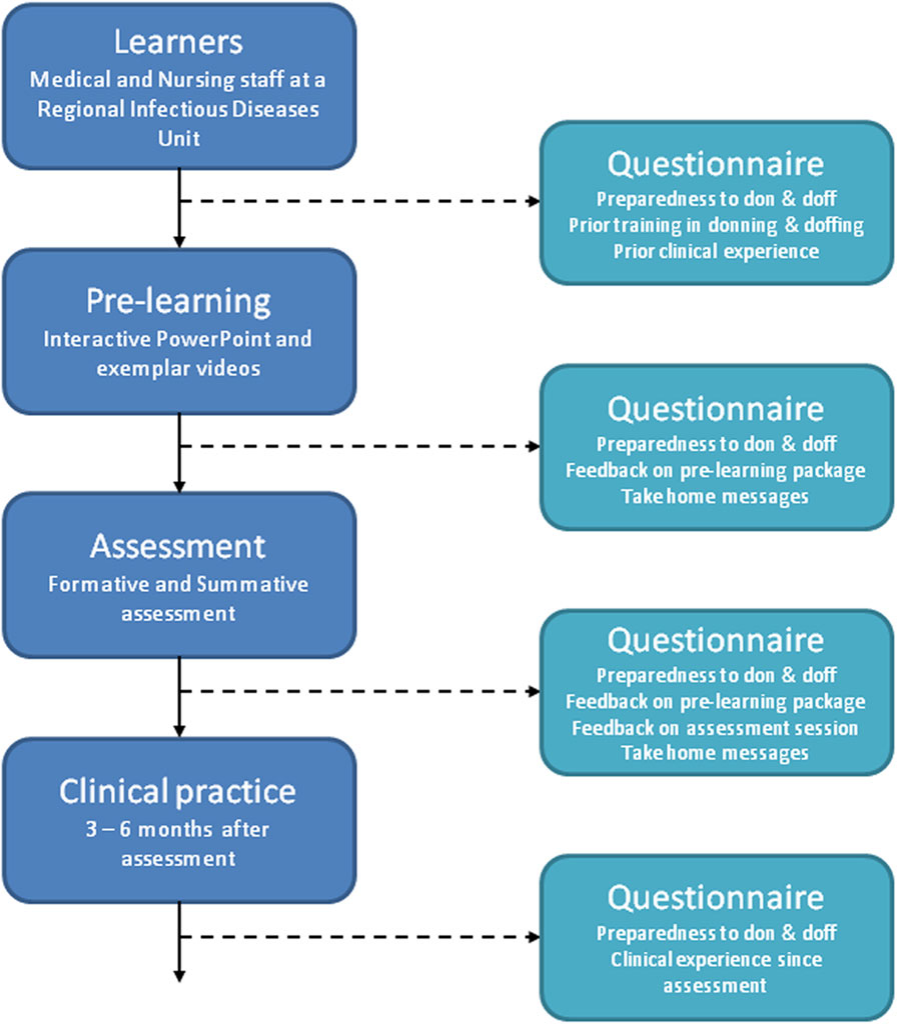
See text for description

#### 0032 Simulation in psychiatry: Developing a bespoke faculty development programme for undergraduate medical educators

##### Kenneth Ruddock, Catriona Neil, Neera Gajree, Catherine Paton

###### NHS Lanarkshire

**Format:** Descriptive Work - Oral Presentations and Short Communications

**Topic:** Faculty Development

***Introduction***

Simulation remains an under-utilised tool in mental health education and training. Within psychiatry, simulation can provide the opportunity for learners to encounter patients presenting with acute psychiatric disorders in a safe, controlled environment.

In NHS Lanarkshire, we developed an immersive simulation course which is now being delivered to all medical students at the University of Glasgow during their clinical psychiatry placement, amounting to around 300 candidates per year.

This has created an acute need for psychiatric simulation-based educators, however, to date, this specialty has been under-represented within the simulation community. We have therefore developed and delivered a bespoke faculty development programme for psychiatrists in the West of Scotland.

***Description***

Due to the significant differences in delivering simulation in psychiatry compared to other medical specialties, we decided a standalone faculty development programme was required.

Training was advertised to psychiatry specialty registrars and consultants working across the West of Scotland and prospective faculty were invited to initially observe an undergraduate simulation course. Our faculty development has been aligned to NHS Scotland’s Clinical Skills Network’s training programme; course candidates are required to complete an e-learning module which provides an introduction to simulation and relevant educational theory.

Following this introduction, prospective faculty attended our one-day faculty development course, which offers candidates the opportunity to develop skills required for simulation, including gaining experience with audio-visual equipment, briefing simulated patients and co-facilitating a debrief utilising the PEARLS framework (Promoting excellence and reflective learning in simulation).

Post-course feedback was obtained via an anonymous questionnaire. From our inaugural course in November 2021, 100% of participants rated the training as ‘excellent’. All respondents ‘agreed’ or ‘strongly agreed’ that the course met its objectives. One candidate described the course as an “excellent opportunity to learn about simulation in psychiatry and how to facilitate”. Following the course, new faculty will continue to develop their skills by facilitating courses alongside experienced faculty.

***Discussion***

Simulation is an emergent area within psychiatry education, with potentially diverse applications in undergraduate and postgraduate training. It is important that simulation-based educators are appropriately trained with their practice grounded in educational theory. Research supports the use of a structured faculty development framework to ensure quality and consistency between educators and simulation centres.

We have developed a bespoke course to train psychiatric simulation-based educators which has been well received by participants. Our course will support the delivery of simulation-based training in psychiatry, providing exciting future opportunities for both learners and faculty.

***Clinical speciality keyword***

Psychiatry; Mental Health

***References/Acknowledgements***

Eppich W, Cheng A. Promoting excellence and reflective learning in simulation (PEARLS): development and rationale for a blended approach to health care simulation debriefing. Society for simulation in healthcare. 2015.

Peterson D, Watts P, Epps C, White M. Simulation faculty development: a tiered approach. Society for simulation in healthcare. 2017.

NHS Scotland Clinical Skills Managed Educational Network (CSMEN). Faculty development: becoming a simulation-based educator. Available from: https://learn.nes.nhs.scot/33268/clinical-skills-managed-educational-network/educational-resources/faculty-development-becoming-a-si

***Ethics Statement***

I have reviewed ethics guidelines and declare these have been followed.

This abstract presents a summary of a newly developed faculty development course. Data presented was collected as an evaluation of the course. No formal research has been undertaken.

#### 0033 Transforming organizational change in community COVID centres: harnessing theoretical informed insitu simulation

##### Gerry Gormley^1^, Sarah O'Hare^1^, Anu Kajamaa^2^, Richard Conn^1^

###### ^1^Queen's University Belfast; ^2^University of Helsinki

**Format:** Descriptive Work - Oral Presentations and Short Communications

**Topic:** Covid-19

***Introduction***

The COVID-19 pandemic has made its impact globally. Within the healthcare ecosystem, many clinical environments had to re-orientate their services in short timeframes. Across the world, the majority of healthcare is community based (e.g. in the NHS >90% in the community setting). Community COVID assessment centres had to be established to safely assess patients with, or suspected as having, COVID-19 infection. Many healthcare facilities had to reorientate their services and built environments. In these centres, systems needed to be set in place to ensure that optimal care could be provided for emergencies situations such as cardiac or respiratory arrest. In Northern Ireland, a number of community COVID centres called upon the simulation community to helps devise and test their emergency systems.

A team of simulationists set about establishing a theoretical informed approach to transform such community based COVID centres to optimise their preparedness for dealing with medical emergencies. The team drew upon Activity Theory (CHAT) and the Transformative Agency Double Stimulation (TADs) model of enhancing organisational change.

***Description***

A team of simulationists engaged with a number of COVID community centres to ensure they had a shared objective – i.e. using in situ simulation to enhance their preparedness in dealing with medical emergencies in these newly form community based COVID centres.

Preliminary site checks / risk assessments were conducted to mitigate any risks with the insitu simulation. A series of insitu simulation scenarios were designed. At a time were no patients were present in the community COVID centres, a range of healthcare workers were recruited to take part in the simulation exercise including doctors, nurses, administrative staff and other professional support staff. The insitu simulation was conducted and recorded using a handheld video camera.

Following the insitu simulation, a workshop was facilitated with all of the healthcare workers. In this workshop, video footage (i.e. mirror data) was used, together with resuscitation guidelines to reflect on the their response in relation to best practice. With the principles of CHAT, discussions were facilitated considering issues, beyond individual performance, that might not otherwise come to light. By identifying such contradictions, participants scoped, agreed, and implemented solutions within the workplace.

One week later a follow up simulation was conducted. This was aimed to test, refine and consolidate the changes implemented. In this descriptive presentation we will share with a range of the adaptions that were devised and implemented.

***Discussion***

Insitu simulation are scenarios conducted in authentic work workplaces. While this adds contextual richness, individual learning has remained in situ simulation’s main emphasis. Our work exemplifies CHAT and TADs as a systemic framework, that can extend the use of in situ simulation, enabling it to drive organizational transformation alongside practitioner development.

***Clinical speciality keyword***

Insitu COVID

***References/Acknowledgements***

Engeström Y. Activity Theory and the Social Construction of Knowledge: A Story of Four Umpires. Organization 2000;7(2):301–310.

***Ethics Statement***

For studies using human or animal subjects:

The authors declare that all procedures followed were in accordance with the ethical standards of the responsible committee on human experimentation (institutional and national ) and with the Helsinki Declaration of 1975 (In its most recently amended version).

Informed consent was obtained from all patients/participants included in the study.

All institutional and national guidelines for the care and use of laboratory animals were followed.

**Table 1 (abstract 0033).** Results of the satisfaction questionnaire applied to participants of the Simulation Training Plan (n= 7)

Item	Strongly agree	Agree
The simulation developed my clinical reasoning skills.	6	1
The simulation developed my clinical decision making ability.	7	0
The simulation enabled me to demonstrate my clinical reasoning skills.	5	2
The simulation helped me to recognise patient deterioration early.	6	1
This was a valuable learning experience.	7	0
The simulation caused me to reflect on my clinical ability.	6	1
The simulation tested my clinical ability.	5	2
The simulation helped me to apply what I learned from the case study.	6	1
The simulation helped me to recognise my clinical strengths and weaknesses.	6	1

#### 0034 U Can't Touch This - A Simulation Intervention to Increase Speaking Up Behaviour for Hand Hygiene

##### Jan Schmutz^1^, Bastian Grande^2^, Hugo Sax^2^

###### ^1^ETH Zurich; ^2^University Hospital Zurich

**Format:** Research Studies - Oral Presentations and Short Communications

**Topic:** Patient Safety / Quality Improvement

***Introduction***

Speaking up in teams, defined as voicing concerns, plays a vital role in avoiding mistakes. Although speaking up has been repeatedly linked with increased performance, team climate or hierarchies prevent employees from voicing their concerns in critical situations. Most studies that have investigated speaking up in medicine are observational studies. Therefore this study aims to conduct and test a speaking-up intervention and investigate its effect on speaking up intention and actual behavior.

***Methods***

We performed two interventions with anesthesia teams in the operating theatre (OR) on a simulation manikin. The same team had to perform induction of anesthesia twice in succession. During each induction, a confederate doctor intentionally violated minor hygiene rules. After the first scenario, one group received an intervention with information on hygiene. The second group received the same hygiene intervention plus a behavioral intervention on Speaking-Up. The speaking-up intervention included reflecting on one's barriers, why one does not speak up, and a simple communication algorithm for speaking up. All scenarios were video recorded. Speaking-up and hygiene violations were coded. Other variables (i.e., self-efficacy of speaking up, psychological safety) were collected using a survey.

***Results & Discussion***

We tested eight 2-person teams in the speaking-up group and 12 teams in the hygiene group. During the pre-measure, participants show no speaking-up behavior. Participants who have undergone a speaking-up intervention show 13% more speaking-up behavior than participants only going through a hygiene intervention (control group). The self-efficacy in relation to speaking up (e.g., “I feel confident that I will speak up in the future”) did not show any change between the groups but was generally on a high level from the start (M=5.5 out of 7). The results suggest that speaking up behavior of healthcare workers can be changed with a short and simple intervention. Long-term effects need further investigation. The results provide important insights for future team training in medicine to increase speaking-up.

***Clinical speciality keyword***

speaking up, teamwork, team training, anaesthesia

***References/Acknowledgements***

NA

***Ethics Statement***

The authors declare that all procedures followed were in accordance with the ethical standards of the responsible committee on human experimentation (institutional and national ) and with the Helsinki Declaration of 1975 (In its most recently amended version).

Informed consent was obtained from all patients/participants included in the study.

All institutional and national guidelines for the care and use of laboratory animals were followed.

**Fig. 1 (abstract 0034).**
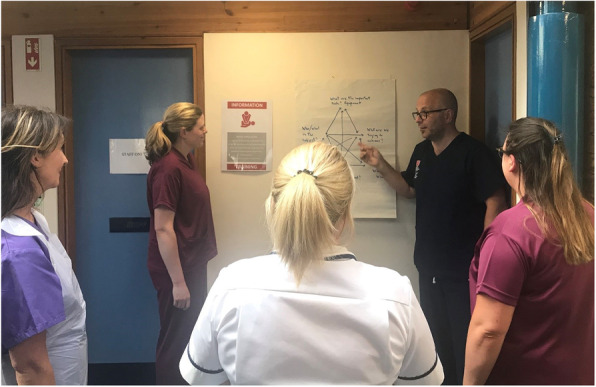
See text for description

#### 0035 Validation and optimization of helmet ventilation (H-CPAP) for patients with moderately severe COVID-19 in low-resource settings

##### John Donahue^1^, Amanda M Chichester^1^, Clinton O Chichester III^1^, Alex Hornstein^2^, Michael Lombardi^3^

###### ^1^University of Rhode Island; ^2^Inventilator LLC; ^3^Lombardi Undersea LLC & Subsalve

**Format:** Research Studies - Oral Presentations and Short Communications

**Topic:** Covid-19

***Introduction***

Helmet continuous positive airway pressure (H-CPAP) is a lifesaving, easy to perform, non-invasive support tool used in COVID-19 patients. It provides necessary respiratory support while significantly reducing SARS-CoV-2 aerosolization to healthcare workers. H-CPAP design and implementation is well-suited to low-resource environments where invasive ventilation equipment, skilled clinicians and ICU beds are in short supply. We sought to establish parameters for safe and effective use of H-CPAP for moderately severe COVID-19 in low resource settings using a TestChest pulmonary simulator.

***Methods***

The capabilities of the TestChest allow for representation of complex breathing patterns and the ability to model patients with worsening COVID-19 pulmonary disease. (Study design is outlined in Figure 1). Initial experiments measured CO2 under conditions of zero gas flow. This was followed by determining minimum range of flow with clinically acceptable EtCO2. To identify the optimal FiO2 and PEEP for the COVID-19 patient, we tested a range of oxygen flow rates with a Venturi circuit.

***Results & Discussion***

Under periods of zero gas flow, helmet CO2 reaches a concentration of 45 mmHg following eight minutes of breathing by a healthy, control patient versus five minutes of breathing by a COVID-19 patient. For extended CO2 rebreathing, the minimum acceptable CO2 concentration was taken to be 7 mmHg or 1% CO2. A fresh gas flow rate ranging between 10-30 liters per minute maintained a low, steady CO2 concentration that ranged from 13mmHg at 10LPM to 5mmHg at 30LPM. The minimum flow to maintain CO2 concentration below 7 mmHg was 25LPM. The Venturi circuit produces high FiO2 and PEEP dependent upon oxygen flow that ranges from 35% FiO2 with PEEP=7 at 10 LPM oxygen, to 48% FiO2 and PEEP=10 at 25 LPM oxygen. When comparing our COVID-19 patient without intervention to our COVID-19 patient with Venturi circuit intervention, an improvement in SpO2. is observed with 25 LPM oxygen delivered by Venturi circuit increasing SpO2 from 79% to 95%. Of importance, the effect of pressure on oxygenation is independent of FiO2. In our less severely affected COVID-19 patient, 14 cmH2O pressure delivered by H-CPAP increased SpO2 from 84% to 94%, despite FiO2 remaining at 21%. These findings suggest less severely affected COVID-19 patients primarily benefit from additional PEEP supplied by H-CPAP. In a low-resource environment, this reduction in oxygen requirement demonstrates benefit for the patient and larger healthcare system. We are presently conducting experiments to determine treatment variables that optimize patient outcomes while minimizing oxygen utilization. We believe our results will provide the foundation to optimize H-CPAP strategies for COVID-19 patients in low-resource settings.

***Clinical speciality keyword***

H-CPAP, COVID-19, NIV, PEEP

***References/Acknowledgements***

Dondorp AM, Hayat M, Aryal D, Beane A, Schultz MJ. Respiratory support in COVID-19 patients, with a focus on resource-limited settings. Am J Trop Med Hyg. 2020;102(6):1191-1197.

Ma X, Vervoort D. Critical care capacity during the COVID-19 pandemic: Global availability of intensive care beds. J Crit Care. 2020;58:96.

***Ethics Statement***

The authors declare that they have followed the guidelines for scientific integrity and professional ethics. The article does not contain any studies with human or animal subjects.

**Fig. 1 (abstract 0035).**
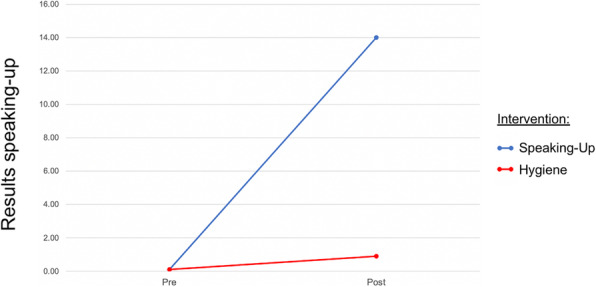
See text for description

#### 0036 What are the competencies required to be successful in the role as a healthcare simulation technician?

##### Adam F. Roche^1^, Claire Condron^2^, Walter Eppich^2^, Paul O'Connor^3^

###### ^1^RCSI; ^2^RCSI, University of Medicine and Health Sciences; ^3^NUI Galway

**Format:** Research Studies - Oral Presentations and Short Communications

**Topic:** Technical Operations

***Introduction***

Healthcare simulation technician (HSTs) are essential to the delivery of simulation-based education. The HST role draws upon a broad range of knowledge, skills and attitude (KSA) competencies. However, due to the neoteric of the job of HST, and the ambiguity surrounding the core responsibilities of the position (Lowther & Armstrong, 2020), it has proved difficult to identify the KSAs required to perform this role successfully. This study aims to identify the KSA competencies required by HSTs.

***Methods***

A mixed methods approach was used in this study. Data was collected from: (1) online searches of HST job descriptions; and (2) semi-structured interviews about the competencies required by HSTs with ten HSTs, ten healthcare simulation educators; and ten healthcare simulation centre managers/director. The data from the job descriptions and interviews was analysed using thematic analysis (Kiger & Varpio, 2020), employing a framework method to guide the coding (Gale et al, 2013).

***Results & Discussion***

A total of 59 competencies were identified from the job descriptions, and 65 competencies from the interviews. This analysis resulted in the identification of nine competency domains: three knowledge domains (technical, clinical, and pedagogic), three skills domains (resourcefulness, pedagogic, team, and technical), and two attitudes domains (professional, and ‘can-do’ mentality).

Conclusions. The identification of the competencies required by HSTs will support the selection of candidates with the attributes that will allow them to be successful in this role, and guide continuous professional development opportunities for current, and future, HSTs.

***Clinical speciality keyword***

Healthcare simulation technician, competencies

***References/Acknowledgements***

1. Lowther, M., & Armstrong, B. (2020). Roles and Responsibilities of a Simulation Technician. 1st edn. Florida: StatPearls

2. Kiger, M., Varpio, L. (2020) Thematic analysis of qualitative data: AMEE Guide No. 131, Medical Teacher, 42:8, 846-854, 10.1080/0142159X.2020.1755030

3. Gale, N.K., Heath, G., Cameron, E., Rashid, S., Redwood, S. (2013). Using the framework method for the analysis of qualitative data in multi-disciplinary health research. BMC Med Res Methodol 13, 117.

10.1186/1471-2288-13-117

***Ethics Statement***

Addressee: Adam Roche

Date: 16/02/2021

Re: CMNHS REC Application

Dear Mr. Roche

We are happy to confirm that your application entitled: “What are the competencies required of healthcare simulation technicians?” has received approval by the College of Medicine, Nursing and Health Sciences Research Ethics Committee.

This is subject to two points that we ask you to update:

1. In the PIL in the section with subheading “Is the study confidential?”, the web address currently reads as www.typist,ie Please correct this.
2. While the title on the confidentiality agreement has been updated, the date of signatures has not, which implies that the signees were not made aware of the change of title. Please correct.

Yours sincerely,

Dr. Sharon Glynn

CMNHS REC co-Chair

**Table 1 (abstract 0036).** KSA competencies and domains derived for job descriptions and interviews

Domain	Competencies	Job descriptions (n = 18)	Interviews (n= 30)	Domain	Competencies	Job descriptions (n = 18)	Interviews (n=30)
**Knowledge competencies**				**Skills competencies cont'd**			
*Technical*	Simulation technology	9; 50%	16; 53%	*Pedagogic*	Instructional	0; 0%	16; 53%
	Information technology (IT)	12; 67%	10; 33%		Role-play	3; 17%	3; 10%
	Audio-visual technology	11; 61%	10; 33%	*Team*	Communication skills	12; 67%	20; 67%
	Communications technology	1; 6%	4; 13%		Teamwork	5; 28%	23; 77%
	Social media	1; 6%	2; 7%		Stakeholder management	10; 56%	9; 30%
*Clinical*	General clinical knowledge	2; 11%	22; 73%		Diplomacy	2; 6%	6; 20%
	Medical equipment	4; 22%	10; 33%		Attentive	3; 17%	4; 13%
	Anatomy and physiology	1; 6%	12; 40%		Leadership	2; 11%	4; 13%
	Medical terminology	5; 28%	6; 20%		Conflict resolution	2; 11%	1; 3%
	Universal precautions	1; 6%	1; 3%	*Technical*	IT skills	14; 78%	18; 60%
*Pedagogic*	Educational principles	3; 17%	13; 43%		General technical skills	10; 56%	11; 37%
	Use of simulation for education	4; 22%	5; 17%		Maintenance	12; 67%	7; 23%
**Skills competencies**					Moulage	8; 44%	11; 37%
*Resourcefulness*	Problem-solving	12; 67%	21; 70%		Research	6; 33%	2; 7%
	Organising	14; 78%	11; 37%		Craft	0; 0%	5; 17%
	Innovative	5; 28%	13; 43%		Videography	1; 6%	2; 11%
	Adaptable	8; 44%	7; 23%	**Attitudes competencies**			
	Planning	8; 44%	5; 17%	*Professional*	Customer-centric	8; 44%	5; 17%
	Procurement	6; 33%	4; 13%		Compliance	7; 39%	4; 13%
	Inventory management	8; 44%	2; 7%		Confidential	6; 33%	5; 17%
	Adept	8; 44%	1; 3%		General professionalism	4; 22%	7; 23%
	Time management	4; 22%	4; 13%		Respectful	1; 6%	8; 27%
	Proactive	0; 0%	8; 27%		Gracious	2; 11%	6; 20%
	Multi-tasking	3; 17%	4; 13%		Inclusive	4; 22%	1; 3%
	Inquisitive	0; 0%	6; 20%		Assertive	0; 0%	5; 17%
	Resilience	4; 22%	1; 3%		Advocacy	0; 0%	4; 13%
	Curriculum development	4; 22%	1; 3%		Responsible	2; 11%	1; 3%
	Strategic development	3; 17%	1; 3%	*Can-do*	Willingness to learn	13; 72%	10; 33%
	Prioritising	2; 11%	1; 3%		Open-minded	2; 11%	12; 40%
	Project management	2; 11%	1; 3%		Self-motivated	7; 39%	4; 13%
	Decision-making	2; 11%	0; 0%		Positivity	3; 17%	8; 27%
	Report writing	2; 11%	0; 0%		Continuous improvement	2; 11%	8; 27%
	Crisis management	1; 6%	1; 3%		Take initiative	6; 33%	3; 10%
	Presentation skills	1; 6%	1; 3%		Autonomous	0; 0%	8; 27%
					Diligence	0; 0%	3; 10%

#### 0037 ‘Lights, camera, action!’: developing educational content for Highly Immersive Virtual Environments (HIVE)

##### Toan Pham^1^, David Hardy^2^, Davina Carr^1^, Paul Hamilton^1^, Gerry Gormley^1^

###### ^1^Queen's University Belfast; ^2^Faculty of Medicine, Health and Life Sciences

**Format:** Descriptive Work - Oral Presentations and Short Communications

**Topic:** New Technologies and Innovation

***Introduction***

Simulation is a potent learning method. Beyond technical-skill development, other dimensions of learning are important including situational awareness. Increasingly, technology is being utilized to enhance context in simulation. One such technology is Highly Immersive Virtual Environments (HIVEs) (1). HIVEs permit learners to be immersed in a physical room and provide an enriched auditory, olfactory and visual context/backdrop (see figure 1); affording learners ‘bodily’ experiences as a scenario unfolds – i.e. a tacit experience that is entangled with place. The possibility of contexts that can be produced in HIVES is potentially limitless. As HIVEs gain traction in simulation, evidence needs to guide how best we optimize such technology in learning. In our project, a cross-discipline team devised a process for development HIVE content.

***Description***

A 6-point plan was co-constructed to guide developing HIVE content:

1. Assemble the team: A cross-discipline team is essential for such initiatives. Not only content experts, but educationalists to underpin the pedagogical intent and technical experts. Most importantly, to engage ‘end users’ (i.e. learners) to ensure the project is learner-centric
2. Define the educational purpose: Central to any effective HIVE content production - is to consider the educational purpose that it will serve. The effectiveness of the HIVE learning activity will hinge on its alignment to the intended educational purpose i.e. ‘Content with intent’
3. Create the story board: Producing a story board of your HIVE content will help refine and enhance its impact. As ever
  important to be mindful of making the scene as timeless and consider diversity in its content
4. Preparation for filming: turn your attention to preparing to capture the content:
  - Location scouting
  - Risk assessment
  - Gaining permissions
  - Schedule a date / time
  - Preparing your AV equipment (including back ups and batteries fully charged!) and any necessary props
  - Planning for ‘other’ factors – including weather for outdoor scenes!
5. On the day:
  Further risk assessment to be carried out and set up the scene
  Gain consent from individuals
  Capture film/audio with the appropriate equipment
  Playback footage to ensure that it is correctly captured – otherwise try again!
  Break down equipment and leave location as you found it
6. Post production
  Review and edit your footage – ensuring it aligns to your educational purpose.
  Trial run content in your HIVE.
  Seek feedback before finalising
  Encourage feedback of how to enhance in the future

***Discussion***

This co-constructed process provides a route map of how to best generate HIVE content, that is grounded in learning. Developing high quality HIVE content will help enrichen learning and allow the sharing of content with colleagues in the simulation community.

***Clinical speciality keyword***

HIVE, simulation

***References/Acknowledgements***

1) de Back, T.T., Tinga, A.M., Nguyen, P. et al. Benefits of immersive collaborative learning in CAVE-based virtual reality. Int J Educ Technol High Educ 17, 51 (2020). 10.1186/s41239-020-00228-9

***Ethics Statement***

The authors declare that they have followed the guidelines for scientific integrity and professional ethics. The article does not contain any studies with human or animal subjects.

**Fig. 1 (abstract 0037).**
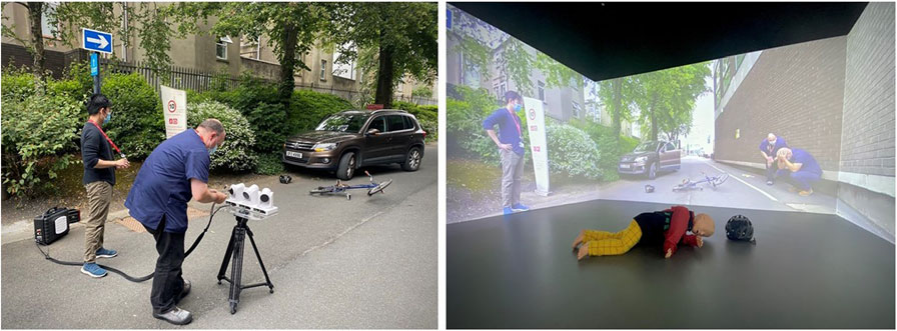
See text for description

#### 0038 “And the Little Child shall lead them” Impact of Virtual Reality Room and Pediatric Emergency Medicine Accelerated Curriculum on Emergency Medicine Residents in a Low Resource Setup

##### Adeel Khatri^1^, Saima Ali^1^, Sama Mukhtar^1^, Syed Ghazanfar Saleem^1^, Jabeen Fayyaz^2^

###### ^1^Indus Hospital and Health Network; ^2^The Hospital for SickKids

**Format:** Research Studies - Oral Presentations and Short Communications

**Topic:** Assessment using Simulation

***Introduction***

Education in Emergency Medicine (EM) is difficult in Low to Middle income (LMIC) countries in view of the rapid turnover of patients and high volumes. EM is in its nascent phase in an LMIC like Pakistan where it was recognized as a separate specialty less than fifteen years ago. (1) The situation is even worse in Pediatric Emergency Medicine (PEM) where the under-five mortality rate is 67.2 deaths for 1000 live births. (2) The need for educational programs in PEM are the need of the day to bridge the gap in knowledge of the EM physicians for better patient outcome.

***Methods***

A two month module based on PEM was administered to the EM residents at the Indus Hospital and Health Network (IHHN). The module was conducted on Zoom in collaboration with PEM faculty from the Hospital for SickKids, Toronto, Canada. An MCQ based pre-test was administered before the start of the module. Classes were held once a week for four hours, with two hours didactic and two hour simulation on Virtual Resuscitation room (VRR). A post-test and a five station, face to face and VRR based Objective Structured Clinical Examination (OSCE) were held at the end of the module.

***Results & Discussion***

Statistical analysis was conducted using STATA 16.0 version. Paired t-test was used to compare the results of pre and post-test for knowledge. The mean score for pre-test (15.94 + 3.73) and post-test (21.89 + 2.02) with a p value of <0.001 showed significant improvement in the resident knowledge post PEM module. The mean score for OSCE was 106.8 + 10.61 out of a total of 135 marks indicating an overall high score for the face to face and virtual PEM simulation. The stratification of the pre and post test results according to the year of residency showed the most improvement in PGY 2 (p 0.04) and the least in PGY1 (0.059), (Table 1).

The improvement in knowledge of the EM residents after the introduction of the PEM module can be further followed up by review of the clinical management of patients and analysis of their outcome. This module can also be scaled up to a certification course and a fellowship program in PEM.

***Clinical speciality keyword***

Pediatric Emergency Medicine, LMIC, Pakistan

***References/Acknowledgements***

1. Razzak JA, Ahmed A, Saleem AF, Nasrullah M. Perceived need for emergency medicine training in Pakistan: a survey of medical education leadership. Emergency Medicine Australasia. 2009 Apr;21(2):143-6.

2. https://data.unicef.org/country/pak/

***Ethics Statement***

This was an educational intervention and was granted Institutional Review Board exemption.

**Table 1 (abstract 0038).** See text for description

	**Pretest**	**Post-Test**	**P-value**
**Overall Mean Score**	15.94 (+3.73)	21.89 (+2.02)	0.001*
	**Stratification of Pre and post test score according to the year of residency**		
	**Pretest**	**Post-Test**	**P-value**
**Year 1**	14.33 (+2.31)	20.33 (+2.88)	0.059
**Year 2**	11.75 (+2.87)	21.75 (+0.50)	0.004*
**Year 3**	18.00 (+2.94)	21.28 (+1.70)	0.037*
**Year 4**	17.40 (+3.36)	23.80 (+1.64)	0.024*

* Significant at <0.05

